# 2009 pandemic H1N1 influenza virus elicits similar clinical course but differential host transcriptional response in mouse, macaque, and swine infection models

**DOI:** 10.1186/1471-2164-13-627

**Published:** 2012-11-15

**Authors:** Jennifer T Go, Sarah E Belisle, Nicolas Tchitchek, Terrence M Tumpey, Wenjun Ma, Juergen A Richt, David Safronetz, Heinz Feldmann, Michael G Katze

**Affiliations:** 1 Department of Microbiology, University of Washington, Seattle, WA, 98195, USA; 2 Washington National Primate Research Center, University of Washington, Seattle, WA, 98195, USA; 3 Department of Diagnostic Medicine/Pathobiology, College of Veterinary Medicine, Kansas State University, Manhattan, KS, 66506, USA; 4 Influenza Division, Centers for Disease Control and Prevention, Atlanta, GA, 30333, USA; 5 Laboratory of Virology, Division of Intramural Research, National Institute of Allergy and Infectious Diseases, National Institutes of Health, Rocky Mountain Laboratories, Hamilton, MT, 59840, USA

**Keywords:** Pandemic, Influenza virus, Genomics, Inflammation, Lipid metabolism, Glucocorticoid receptor, LXR/RXR

## Abstract

**Background:**

The 2009 pandemic H1N1 influenza virus emerged in swine and quickly became a major global health threat. In mouse, non human primate, and swine infection models, the pH1N1 virus efficiently replicates in the lung and induces pro-inflammatory host responses; however, whether similar or different cellular pathways were impacted by pH1N1 virus across independent infection models remains to be further defined. To address this we have performed a comparative transcriptomic analysis of acute phase responses to a single pH1N1 influenza virus, A/California/04/2009 (CA04), in the lung of mice, macaques and swine.

**Results:**

Despite similarities in the clinical course, we observed differences in inflammatory molecules elicited, and the kinetics of their gene expression changes across all three species. We found genes associated with the retinoid X receptor (RXR) signaling pathway known to control pro-inflammatory and metabolic processes that were differentially regulated during infection in each species, though the heterodimeric RXR partner, pathway associated signaling molecules, and gene expression patterns varied among the three species.

**Conclusions:**

By comparing transcriptional changes in the context of clinical and virological measures, we identified differences in the host transcriptional response to pH1N1 virus across independent models of acute infection. Antiviral resistance and the emergence of new influenza viruses have placed more focus on developing drugs that target the immune system. Underlying overt clinical disease are molecular events that suggest therapeutic targets identified in one host may not be appropriate in another.

## Background

In 2009 a novel H1N1 influenza virus emerged and rapidly spread worldwide [1]. Clinical disease among affected individuals ranged from mild infection to more severe pneumonia associated with acute respiratory distress syndrome [2,3]. A recent study estimates that over 284,000 deaths occurred globally within the first year of the pandemic [4]. Highly pathogenic influenza virus infection is characterized by a powerful and potentially destructive immune response [5,6]. Enhanced cytokine production has been observed in cynomolgus macaques infected with 2009 pandemic H1N1 influenza (pH1N1) virus compared to former seasonal H1N1 influenza virus [7,8], though pH1N1 virus does not precipitate a ‘cytokine storm’ seen with highly pathogenic influenza viruses (reviewed in [9]).

The pH1N1 virus has been intensively studied in mice, macaques and swine, among other animal models of influenza infection. In the absence of prior host adaptation, inoculation of mice with pH1N1 virus generally results in nonlethal infection that is resolved around day 8 post-infection. There is low morbidity (<10% total body weight) and moderate bronchiolitis observed in the lung, despite efficient viral replication throughout infection [10]. Alteration of viral PB1-F2 and NS1 proteins marginally impacted viral pathogenesis [11,12] and did not affect viral control of innate immune responses during pH1N1 infection in mice [13]. Cynomolgus macaques infected with pH1N1 virus have shown diffuse alveolar damage, more severe pulmonary lesions, and efficient replication of the virus in the lungs compared to seasonal H1N1 influenza virus [7,8,14]. Swine infected with pH1N1 virus develop mild respiratory disease characterized by coughing, sneezing, and acute bronchiolitis, with active replication in the lung [15]. Recent studies have shown that the Eurasian-origin NA and M genes of pH1N1 virus are important for transmission among swine [16] and can also confer transmission in ferrets [17] and guinea pigs [18].

Differential gene expression induced by pH1N1 virus compared to concurrent human or swine influenza viruses has been investigated in mice, macaques and swine by microarray. In mice, the transcriptional program suggests that regulation of lipid metabolism genes plays a protective role, differentiating non-lethal wild-type and lethal mouse-adapted pH1N1 virus infection [10]. Macaques infected with clinical pH1N1 isolates from Mexico showed increased expression of NFκB signaling molecule genes, cytokine and chemokine genes, as well as antigen presentation pathway genes compared to seasonal H1N1 influenza virus [7]. In a separate study, macaques infected with pH1N1 virus also showed enhanced expression of antiviral and interferon (IFN)-regulated genes, such as IFIT2 and ISG15, compared to seasonal influenza virus [14]. Transcriptomic analysis of swine host responses to pH1N1 virus revealed pronounced inflammatory response gene expression accompanied by increased expression of PPARG-associated lipid metabolism genes compared to the 1918-like classical swine influenza A/swine/Iowa/15/1930 (H1N1) virus [15]. In contrast to *in vivo* models, transcriptomic profiling of infected type I alveolar epithelial cells showed comparable IFN-mediated antiviral and cytokine responses to pH1N1 and seasonal H1N1 influenza viruses [19].

Performing a comparative transcriptomic analysis across multiple model systems has revealed conserved responses during influenza infection, as demonstrated in a recent study by McDermott et al. that used multivariate modeling approaches to identify similarities in transcriptional responses to H5N1 virus in the lungs of mice and macaques, and human lung epithelial cells [20]. To gain further insight into host responses to pH1N1 virus during acute infection, we examined lung gene expression from mice, macaques and swine infected with 2009 pandemic H1N1 influenza A/California/04/2009 (CA04) virus and compared the transcriptional response in each host. Our goals were to identify shared or differential gene expression patterns across species and to infer potential regulators mediating these changes during acute infection. Even though CA04 virus elicited a similar clinical outcome in each of these species, we found significant differences in the expression of inflammatory and lipid metabolism genes, likely impacted by nuclear hormone receptor signaling complexes including LXR/RXR that is known to regulate cholesterol homeostasis during inflammation. A greater understanding of the differences in acute responses from different hosts is important because it will aid in the design of tailored immunotherapies to influenza virus.

## Results and discussion

We have previously shown 2009 pandemic H1N1 influenza A/California/04/2009 (CA04) virus efficiently replicates in the lung of mice and swine, inducing expression of pro-inflammatory genes and causing acute bronchiolitis [10,15]. Infection of macaques with CA04 virus is marked by productive virus replication in the respiratory tract and moderately severe clinical symptoms peaking on day 6 post-infection (p.i.), with resolution by day 14 p.i. (^a^Safronetz and Feldmann, personal communications). Here, we examined mouse, macaque and swine responses to CA04 virus by microarray to further investigate transcriptional changes during acute pH1N1 infection. Mice were inoculated with 10^6^ PFU virus and three animals were euthanized on days 1, 3, and 5 p.i. These three time points were selected for studying acute phase responses in the mouse. Cynomolgus macaques were inoculated with a total infectious dose of 7×10^6^ TCID_50_ and two animals were euthanized on days 1 and 6 p.i. Day 1 macaque lung samples were collected for studying early host immune responses corresponding to early disease progression and day 6 lung samples were chosen for maximum pathology. Swine were inoculated intratracheally with 10^6^ TCID_50_/animal and five animals were euthanized on days 3, 5 and 7 p.i. Swine lung samples were collected on days 3 and 5 to examine acute phase responses corresponding to maximal virus shedding and day 7 lung samples were chosen for recovery phase. Due to differences in the timing and kinetics of acute phase responses in each animal model, we examined gene expression changes within each host and then compared across species.

Viral mRNA expression was measured in each sample to verify CA04 infection prior to microarray analysis (Additional file 1: Figure S1). In general, viral mRNA levels correlated with viral titers measured in the lung for each species. In mice, viral mRNA expression averaged between 3.6 – 4.5 log_10_RQ on day 1 to 5 p.i., corresponding to average lung virus titers between 5.2 – 6.1 log_10_ PFU/g of tissue on day 1 to 5 p.i. [10]. In macaques, average viral mRNA expression was highest on day 1 and decreased by day 6 p.i., which was also reflected in the virus lung titers that reached around 10^7^ and 10^4^ TCID_50_(log_10_)/g on days 1 and 6 p.i., respectively (^a^Safronetz and Feldmann, personal communications). In swine, viral mRNA levels paralleled virus shedding (from days 3 to 5 p.i.), and the decreased viral mRNA expression observed on day 7 p.i. was concomitant with resolution of infection in these animals [15]. Quantitation of viral mRNAs in infected samples used for microarray allowed us to correlate host gene expression changes with relative infection levels.

### Gene expression analysis of mouse, macaque and swine lung infected with CA04 virus

Gene expression was profiled using species-specific commercial oligonucleotide arrays. Each array contained a different set of transcripts, with greater redundancy for macaque and swine compared to mouse. There are a total of 43,379 probes represented on the Mouse Whole Genome Gene Expression Microarray, a total of 20,217 probes represented on the Rhesus Macaque Gene Expression Microarray, and a total of 47,813 probes represented on the Porcine Gene Expression Microarray V1. Due to differences in gene annotation across species, differential gene expression analysis was performed for the transcripts associated with 4118 unique genes common to all three arrays using Ensembl gene identifiers associated with each array probe. This strategy accounted for differences in the number of probes with annotated transcripts and reduced the likelihood of falsely identifying differences in gene expression due to gaps in annotation or gene representation. Within this set of genes, there was a large representation of genes associated with Cell Death, Cancer, and Cellular Growth and Proliferation functional categories, as well as genes associated with Glucocorticoid Receptor Signaling, IL-12 Signaling and Production in Macrophages, and Acute Phase Response Signaling canonical pathways.

We investigated the functional classes of underrepresented gene sets present on each specie-specific array, as the focus on unique genes common to all three arrays may introduce bias in our analyses. There was enrichment of Molecular Mechanisms of Cancer, Axonal Guidance Signaling and G-Protein Coupled Receptor Signaling canonical pathways, and Gene Expression functional annotations related to transcription, organismal death and abnormal morphology of cells. Data integration and interpretation with a cross-species transcriptomic analysis brings its own challenges and although complete physical maps have been developed for mouse, macaque and swine genomes to support genome sequencing and comparative genomics, functional annotation to date is mostly based on human, mouse, and rat literature. As annotation improves, particularly for less characterized species such as swine, we will likely be able to more fully understand host responses to influenza virus using microarray and next-generation sequencing technologies.

**Figure 1:**
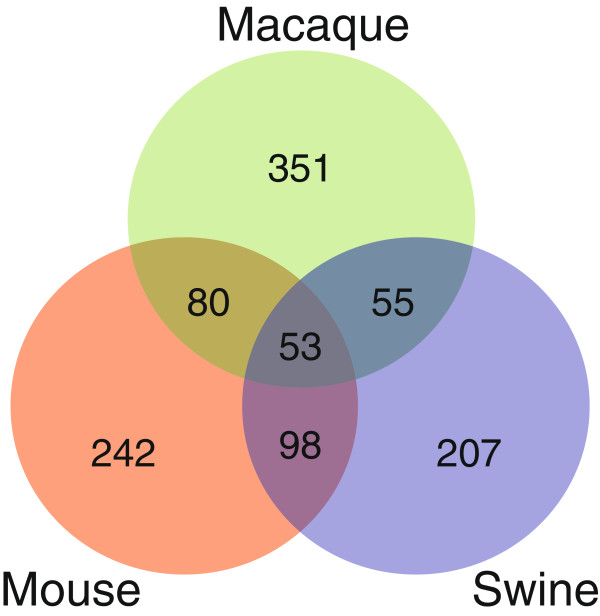
**Comparison of significant host genes differentially regulated during CA04 infection in each species.** Venn diagram showing the overlap between mouse (red), macaque (green) and swine (blue) DE gene sets.

Of the 4118 unique genes common to all three species arrays, we identified a total of 696 differentially expressed (DE) genes in mice, 771 DE genes in macaque, and 611 DE genes in swine that significantly changed in response to CA04 virus on at least one day (Student’s t-test *P* < 0.05, average fold-change ≥ 2). The Venn diagram shown in Figure 1 illustrates the overlap of CA04 virus-induced DE genes among mouse, macaque and swine infection models, and only 53 genes were differentially expressed in all three species. Functional analysis of each specie DE gene set revealed significant enrichment of genes associated with Acute Phase Response Signaling, LXR/RXR Activation, and Atherosclerosis Signaling canonical pathways in infected mice, VDR/RXR Activation, Hematopoiesis from Pluripotent Stem Cells, and Communication between Innate and Adaptive Immune Cells canonical pathways in infected macaques, and LXR/RXR Activation, Nitrogen Metabolism, and Antigen Presentation Pathway canonical pathways in infected swine (Table 1). The genes associated with each canonical pathway shown in Table 1 are reported in Additional file 2.

**Table 1:** Canonical Pathways enriched in mouse, macaque and swine CA04 infection models

**Infection model**	**Canonical pathway**	**B-H *p-*value**	**mol**^**a**^**/mol**^**b**^
***Mouse***	Acute Phase Response Signaling	2.34E-10	28/177
	LXR/RXR Activation	2.34E-10	26/136
	Atherosclerosis Signaling	3.47E-09	22/129
	Hepatic Fibrosis / Hepatic Stellate Cell Activation	6.31E-07	21/147
	IL-12 Signaling and Production in Macrophages	1.12E-06	20/155
	Complement System	4.07E-06	10/35
	Type I Diabetes Mellitus Signaling	5.50E-06	17/121
	Nitrogen Metabolism	8.51E-05	9/120
	Clathrin-mediated Endocytosis Signaling	1.30E-04	20/195
	Dendritic Cell Maturation	1.70E-04	18/185
***Macaque***	VDR/RXR Activation	3.63E-03	13/81
	Hematopoiesis from Pluripotent Stem Cells	5.25E-03	10/64
	Communication between Innate and Adaptive Immune Cells	2.75E-02	12/109
	Type I Diabetes Mellitus Signaling	3.16E-02	13/121
	Altered T Cell and B Cell Signaling in Rheumatoid Arthritis	3.80E-02	11/92
	Gα12/13 Signaling	3.80E-02	13/127
	Neuroprotective Role of THOP1 in Alzheimer's Disease	3.80E-02	7/54
	Hematopoiesis from Multipotent Stem Cells	3.80E-02	4/12
	Primary Immunodeficiency Signaling	3.80E-02	8/63
	Pyrimidine Metabolism	3.80E-02	14/215
***Swine***	LXR/RXR Activation	1.15E-07	20/136
	Nitrogen Metabolism	9.33E-04	8/120
	Antigen Presentation Pathway	9.33E-04	8/40
	Communication between Innate and Adaptive Immune Cells	9.33E-04	12/109
	Hepatic Fibrosis / Hepatic Stellate Cell Activation	9.33E-04	15/147
	Type I Diabetes Mellitus Signaling	9.33E-04	13/121
	Primary Immunodeficiency Signaling	9.33E-04	9/63
	Role of Pattern Recognition Receptors in Recognition of Bacteria and Viruses	9.33E-04	12/106
	TREM1 Signaling	9.33E-04	9/66
	Atherosclerosis Signaling	1.12E-03	13/129

Ingenuity Pathway Analysis was used to determine the top 10 Canonical Pathways. Benjamini-Hochberg (B-H) Multiple Testing Correction *p*-value was used to rank the significance associated for each pathway.

^a^ Number of molecules differentially expressed in the Canonical Pathway.

^b^ Total number of molecules in the annotated Canonical Pathway.

**Figure 2:**
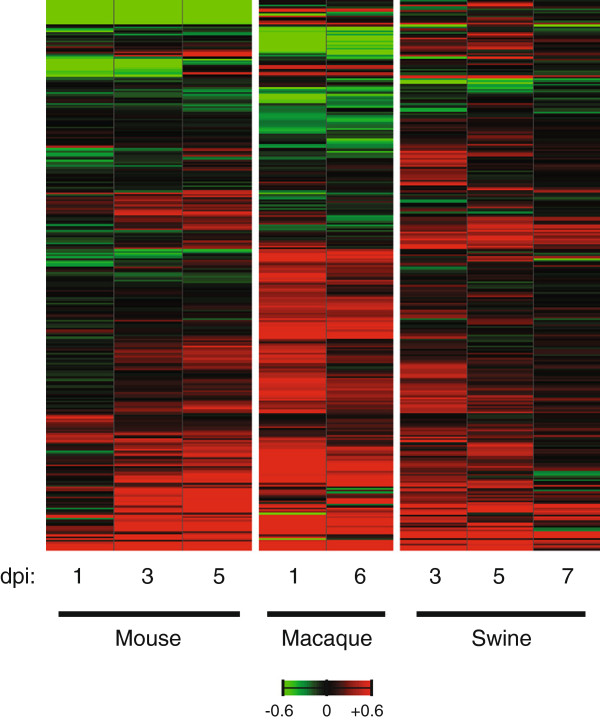
**Heatmap of inflammatory response genes across all three species infected with CA04 virus.** Average log_10_(ratio) expression of 319 DE inflammatory response genes induced by CA04 virus. In mice and swine, infected lung gene expression is referenced to specie-matched mock at each time point. In macaques, infected lung gene expression is relative to an uninfected lung reference pool at each time point. Red indicates expression was increased relative to the control reference and green indicates that expression was decreased relative to the control reference. Saturation is 4-fold.

Inflammatory Response was significantly enriched in all three CA04 infection models, with predicted increased activation in macaques and swine (corrected z-scores of 3.437 and 4.02, respectively) (Additional file 3: Table S2). A similar number of DE inflammatory response genes characterized CA04 infection in mice, macaques and swine (175 DE genes in mice; 166 DE genes in macaques; and 160 DE genes in swine), though the kinetics of the host response was distinct in each species (Figure 2). In general, mice showed an increase in gene expression as infection progressed, whereas the majority of these genes were consistently upregulated or downregulated on days 1 and 6 p.i. in CA04 virus-infected macaques. In contrast to mice and macaques, swine exhibited enhanced gene expression on days 3 and 5 p.i. and the host response tapered as the infection resolved. The dissimilarity in the kinetics of gene expression across species may be in part due to differences in virus replication and timing of acute phase responses. Therefore, a more complete kinetic time course, including a range of inoculation doses, would be necessary to fully appreciate the impact of differences in acute phase responses to pH1N1 virus infection.

### Differential inflammatory response gene expression distinguishes CA04 virus infection in mice, macaques and swine

**Figure 3:**
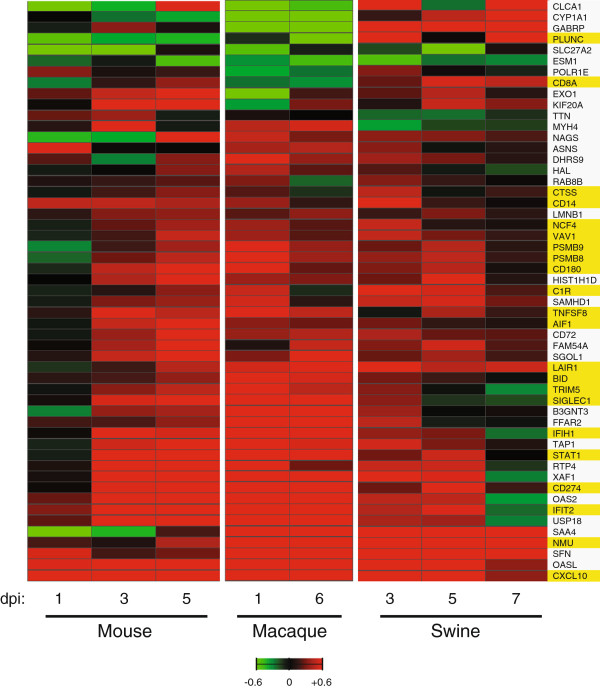
**Heatmap of 53 genes commonly differentially regulated across all three species infected with CA04 virus.** Average log_10_(ratio) expression of 53 DE genes induced by CA04 virus. In mice and swine, infected lung gene expression is referenced to specie-matched mock at each time point. In macaques, infected lung gene expression is relative to an uninfected lung reference pool at each time point. Red indicates expression was increased relative to the control reference and green indicates that expression was decreased relative to the control reference. Saturation is 4-fold. A total of 22 DE genes associated with inflammatory response are highlighted on the right; yellow denotes positive association.

Among the inflammatory response genes there was representation of Acute Phase Response Signaling, LXR/RXR Activation, and Dendritic Cell Maturation canonical pathways in infected mice, Role of NFAT in Regulation of the Immune Response, Role of Macrophages, Fibroblasts and Endothelial Cells in Rheumatoid Arthritis, and G-Protein Coupled Receptor Signaling canonical pathways in infected macaques, and enrichment of inflammatory response genes associated with Glucocorticoid Receptor Signaling, Dendritic Cell Maturation, and LXR/RXR Activation canonical pathways in infected swine. When we examined the 53 genes common to mice, macaques and swine, we noted the majority of genes were upregulated in response to infection in mice on days 3 and 5 p.i., in macaques on days 1 and 6 p.i., and in swine on days 3 and 5 p.i. (Figure 3). Inflammatory response genes (highlighted yellow) included interferon (IFN) signaling molecule, STAT1, IFN-regulated antigen presentation and immunoproteasome components, TAP1, PSMB8 and PSMB9, Toll-like receptor 4 coreceptor, CD14, and antiviral effectors, IFIH1 and IFIT2, previously shown to play essential roles in innate immune response to influenza infection. We also found several leukocyte-specific genes commonly differentially expressed during CA04 infection. Members of the immunoglobulin receptor superfamily upregulated across species included CD72, CD274 (also known as programmed cell death ligand 1), and CD180, a TLR homologue expressed on B cells, macrophages and dendritic cells. Macrophage-restricted receptor, SIGLEC1, neutrophil factors, PLUNC and NCF4, and T lymphocyte coreceptor, CD8A, were also differentially expressed during CA04 infection in each species (Figure 3, Additional file 4: Table S3).

Several models show an association between enhanced immune cell infiltrate and severe lung immunopathology. Excessive macrophage and neutrophils are observed in the lung of mice following H5N1 and 1918 infection [21], and in pregnant animals infected with pH1N1 virus [22]. In mice, macaques, and swine infected with CA04 virus, leukocyte and lymphocyte responses were evident in all three species based on immune cell-specific gene expression changes detected in the lung, though the gene expression patterns varied across species. For example, there was greater upregulation of macrophage factor, SIGLEC1, observed in mice (days 3 and 5 p.i.) and macaques (days 1 and 6 p.i.) compared to swine on day 3 p.i. Enhanced expression of leukocyte associated immunoglobulin-like receptor 1, LAIR1, was observed in macaques and swine infected with CA04 virus, as compared to infected mice. In contrast to mice and macaques, swine exhibited strong expression of neutrophil factor, PLUNC, which we noted was downregulated during infection in the other species (Figure 3). These results reflect a rapid inflammatory shift in the lungs of mice, macaques, and swine during CA04 infection that involves different immune cell responses, but unlike H5N1 and 1918 viruses, these immune cell responses do not cause immunopathology. A cross-species transcriptomic comparison of the host response to H5N1 and 1918 viruses would be necessary to further explore the potential role of specific immune cell responses to influenza pathogenesis.

### CA04 infection impacts glucocorticoid receptor signaling differently across species

We further sought to evaluate CA04 virus-induced host responses that were unique to each species by investigating non-overlapping gene sets shown in Figure 1. The most significant canonical pathways represented in each of these three gene sets are summarized in Additional file 5: Figure S2. The 207 DE gene set unique to swine was enriched for genes associated with Role of JAK1 and JAK3 in γc Cytokine Signaling canonical pathway that included JAK3 and STAT5A genes. Differential regulation of JAK3/STAT5 signaling in CA04 virus-infected swine may impact the homeostasis and activation of peripheral T lymphocytes. For example, STAT5A/B defects results in enhanced apoptosis of T lymphocytes in mice [23]. Differential expression of JAK3 and STAT5A genes in swine during CA04 infection may also suggest a balance between inflammatory host defenses and glucocorticoid receptor (GR)-mediated cellular growth and survival. Closer inspection of each species inflammatory response DE gene set showed noteworthy representation of GR Signaling related genes, such as A2M, FGG, HSPA5, IL4, and MAPK13 genes in mice, FKBP4, JAK1, NCOA2 (also known as GRIP-1), and NRAS genes in macaques, and HSPA4 (also known as HSP70) in addition to JAK3, and STAT5A genes in swine. Alpha-2-macroglobulin (A2M) identified by mass spectrometry in human saliva was found to exhibit antiviral activity against pH1N1 virus [24].

**Figure 4:**
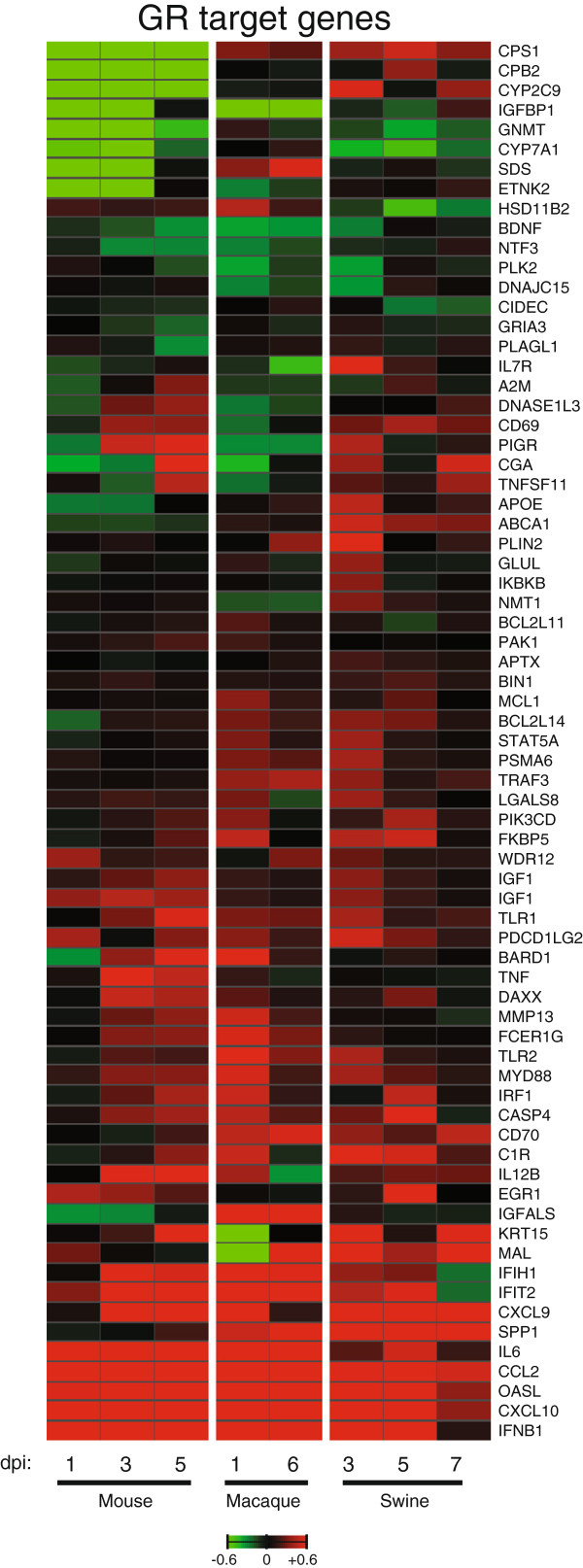
**Glucocorticoid receptor target genes are differentially expressed in response to CA04 virus.** Average log_10_(ratio) expression of downstream GR target genes induced by CA04 virus. In mice and swine, infected lung gene expression is referenced to specie-matched mock at each time point. In macaques, infected lung gene expression is relative to an uninfected lung reference pool at each time point. Red indicates expression was increased relative to the control reference and green indicates that expression was decreased relative to the control reference. Saturation is 4-fold.

Since GR signaling appears to play a role during CA04 infection in mice, macaques, and swine, we used a transcription factor (TF) analysis strategy that combined transcriptomic and DNA sequence information to investigate GR and other potential regulators controlling host responses during CA04 infection (described in Methods). As summarized in Table 2, we found predicted inhibition of hepatocyte nuclear factor 1alpha, HNF1A, in infected mice on all three days, consistent with our previous report [10], and predicted activation of pro-inflammatory regulators, STAT1, NFκB, and IRF7 in at least two species. Examination of downstream gene targets of GR determined using IPA Upstream Regulator Analysis showed differences in expression across species with more pronounced transcriptional changes observed in mice on days 3 and 5 p.i. and on the early time points in macaques and swine (Figure 4). Through a DNA sequence-based approach, we assessed the representation of DNA-binding motifs in the promoter region of DE genes targeted by HNF1A and STAT1 using DNA sequences from each species genome and regulator DNA-binding preferences, modeled as affinity Position Weight Matrices (PWM) (Additional file 6: Figure S3).

**Table 2:** Upstream Regulator analysis of mouse, macaque and swine CA04 infection models

**Upstream regulator**	**Molecule type**	***p*-value of overlap**	**Regulation z-score (dpi)**	**Predicted regulator status**
***Mouse***				
STAT3	transcription regulator	1.68E-17		
STAT1	transcription regulator	2.80E-17	2.747 (3); 2.124 (5)	Activated
PPARA	ligand-dependent nuclear receptor	1.12E-15		
NFκB (complex)*	complex	2.37E-13	2.463 (3); 2.787 (5)	Activated
JUN	transcription regulator	6.74E-13	2.072 (5)	Activated
YY1	transcription regulator	4.65E-12		
SMAD3	transcription regulator	5.13E-12		
IRF7	transcription regulator	3.52E-11	2.994 (3); 2.532 (5)	Activated
IRF1	transcription regulator	4.14E-11	2.125 (3)	Activated
HNF1A*	transcription regulator	5.01E-11	-3.166 (1); -3.743 (3); -3.035 (5)	Inhibited
***Macaque***				
IRF1	transcription regulator	2.16E-10	2.547 (1)	Activated
SPI1	transcription regulator	4.74E-08	2.490 (1)	Activated
TRIM24	transcription regulator	2.43E-07	-3.395 (1, 6)	Inhibited
IRF7	transcription regulator	4.98E-07	4.284 (1, 6)	Activated
STAT3	transcription regulator	4.31E-06	2.096 (1, 6)	Activated
STAT1	transcription regulator	1.15E-05	3.232 (1); 3.57 (6)	Activated
JUN	transcription regulator	1.57E-05		
IRF8	transcription regulator	7.08E-05		
VitaminD3-VDR-RXR	complex	7.75E-05		
ELF1	transcription regulator	9.71E-05		
***Swine***				
STAT1*	transcription regulator	6.54E-14	2.823 (3); 3.157 (5)	Activated
TRIM24	transcription regulator	2.59E-11		
IRF1*	transcription regulator	7.54E-11		
PPARA	ligand-dependent nuclear receptor	2.34E-09	-2.001 (3)	Inhibited
STAT3	transcription regulator	2.35E-09		
NFκB complex	complex	2.48E-09	3.01 (5)	Activated
IRF2*	transcription regulator	7.64E-09		
IRF7	transcription regulator	1.52E-08		
JUN	transcription regulator	4.38E-08		
NFKB1	transcription regulator	2.32E-07	2.136 (7)	Activated

Ingenuity Pathway Analysis was used to determine the top 10 Upstream Regulators. The *p*-value of overlap was used to rank the significance associated for each Upstream Regulator. The *p*-value indicates the significance of the overlap between the genes targeted by the upstream regulator in the IPKB database and the experimental dataset. Genes with an apteryx signify regulators that were identified as significantly enriched (*P* < 0.0001) by PSCAN. Z-scores for predicted upstream regulators (|z|>2) in each species and at each time point are shown. Z > 2 predicts activation of the upstream regulator. Z < -2 predicts inhibition of the upstream regulator.

We have identified enriched TFs and targets from our dataset; however, the analyses reported here coordinately investigate the expression and sequence-specific TF DNA binding sites of the DE genes, and it does not investigate the combinatorial effects of multiple factors binding to the genomic regions or molecular determinants of transcriptional responses such as receptor ligation or histone acetylation that can collectively contribute to distinct transcriptional profiles [25]. In addition, restriction of the search space to -450 to +50 nucleotides relative to the transcription start site (TSS) can preclude the identification of more distal TFs effecting expression, which may explain why GR is not identified as a predicted regulator (Table 2), although many target genes are differentially expressed in the data set (Figure 4). Future investigations of GR-mediated host responses to CA04 virus would need to take into account these considerations as well as the physical interactions between GR and STAT5, for example, and chromatin modifications known to occur [26] to provide a more comprehensive view of transcriptional regulation during CA04 infection across different species.

### CA04 virus significantly alters expression of genes involved in cholesterol homeostasis in mice and swine, and vitamin D receptor genes in macaques

**Figure 5:**
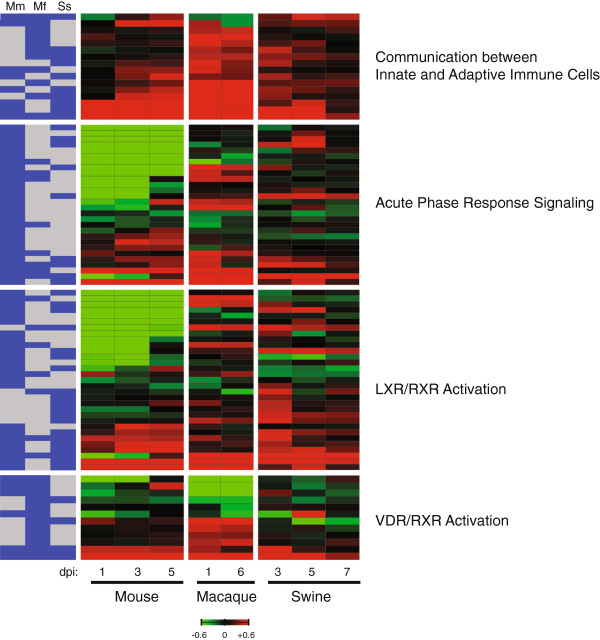
**Immune genes exhibit unique expression profiles in mice, macaques, and swine infected with CA04 virus.** Average log_10_(ratio) expression of DE genes induced by CA04 virus. In mice and swine, infected lung gene expression is referenced to specie-matched mock at each time point. In macaques, infected lung gene expression is relative to an uninfected lung reference pool at each time point. Red indicates expression was increased relative to the control reference and green indicates that expression was decreased relative to the control reference. Saturation is 4-fold. There are a total of 16 DE genes represented for Communication between Innate and Adaptive Immune Cells, 28 DE genes represented for Acute Phase Response Signaling, 31 DE genes represented for LXR/RXR Activation, and 12 DE genes represented for VDR/RXR Activation. The association of each DE gene within a given species for each of the represented Canonical Pathways is shown on the left; blue denotes differential expression and gray denotes no differential expression. The following abbreviations are used: *Mm*, *Mus musculus* (mouse); *Mf*, *Macaca fascicularis* (macaque); and *Ss*, *Sus scrofa* (swine).

Vitamin D receptor (VDR) and liver X receptor (LXR) are ligand-activated transcription factors of the nuclear receptor superfamily that heterodimerize with retinoid X receptor (RXR) to modulate an array of immune and metabolic programs (reviewed in [27,28]). Perturbation of RXR-mediated signaling pathways have shown unique roles for VDR in macrophage responses to *Mycobacterium tuberculosis*[29], and LXR in macrophage responses to *Listeria monocytogenes*[30]. In mice and swine infected with CA04 virus, we found LXR/RXR Activation was among the most significant canonical pathways differentially regulated during acute infection, while VDR/RXR Activation was the most significant canonical pathway differentially regulated in CA04 virus-infected macaques (Table 1). As shown in Figure 5, gene expression profiles of LXR/RXR and VDR/RXR associated pathway molecules, as well as genes enriched for Communication between Innate and Adaptive Immune Cells and Acute Phase Response Signaling canonical pathways strongly emphasize the distinct nature of the host response to CA04 virus in these three animal models.

**Figure 6:**
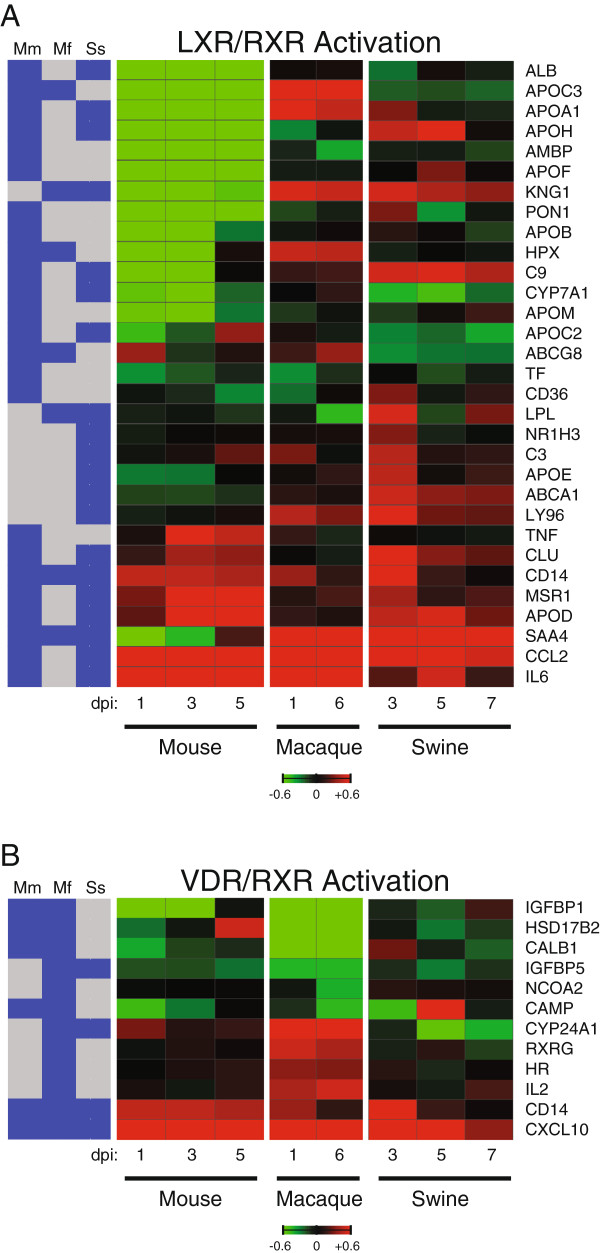
**CA04 virus induces different RXR signaling-mediated responses in each species.A**) Average log_10_(ratio) expression of 31 LXR/RXR DE genes induced by CA04 virus. In mice and swine, infected lung gene expression is referenced to specie-matched mock at each time point. In macaques, infected lung gene expression is relative to an uninfected lung reference pool at each time point. Red indicates expression was increased relative to the control reference and green indicates that expression was decreased relative to the control reference. Saturation is 4-fold. **B**) Average log_10_(ratio) expression of 12 VDR/RXR DE genes induced by CA04 virus. In mice and swine, infected lung gene expression is referenced to specie-matched mock at each time point. In macaques, infected lung gene expression is relative to an uninfected lung reference pool at each time point. Red indicates expression was increased relative to the control reference and green indicates that expression was decreased relative to the control reference. Saturation is 4-fold. The association of each DE gene within a given species is shown on the left; blue denotes differential expression and gray denotes no differential expression. The following abbreviations are used: *Mm*, *Mus musculus* (mouse); *Mf*, *Macaca fascicularis* (macaque); and *Ss*, *Sus scrofa* (swine).

Macrophages have been shown to increase in mouse lungs following influenza infection and these cells are specific targets of viral infection [21,22]. In response to CA04 virus, we observed a number of genes related to macrophages and with roles in lipid metabolism, such as cholesterol efflux, when examining LXR/RXR Activation in mice and swine. In mice, there was marked decreased expression of apolipoprotein genes, including APOE and APOA1, and moderate downregulated expression of macrophage-specific genes involved in recognition of oxidized lipids, such as macrophage scavenger receptor genes MSR1 (SR-A1) and CD36 (Figure 6, top panel; Additional file 7: Table S4). The ABCA1 gene, known for its role in cholesterol transport, and the LPL gene, involved in lipid catabolism, were also downregulated in mice. In contrast to mice, swine showed strong upregulation of these genes associated with cholesterol efflux on day 3 p.i. Previous studies have shown a decrease in ABCA1 mRNA expression in LXR-activated primary macrophages via an IRF3-dependent mechanism following influenza infection [31]. Here, we observe that ABCA1 and other genes associated with cholesterol efflux are significantly downregulated in response to CA04 infection in mice, but upregulated in CA04 virus-infected swine lung, suggesting that mice may be more efficient at suppressing cholesterol efflux, and in turn, possibly preventing the formation of lipid-laden macrophages (also known as foam cells). Foam cells are the product of inflammatory responses ranging from atherosclerosis (reviewed in [32]) to infection by *Chlamydia pneumonia*[33] and *Toxoplasma gondii*[34]. While the link between influenza-induced innate immunity and cholesterol metabolism is poorly defined, our findings suggest a role for LXR/RXR signaling during CA04 infection and indicate that this cellular pathway is impacted differently in two distinct hosts.

In human macrophages and respiratory epithelial cells, vitamin D plays a role in innate immune responses by increasing TLR coreceptor, CD14, and stimulating expression of antimicrobial peptides, such as cathelicidin during respiratory infection [35,36]. Vitamin D has also been investigated in epidemiological studies of reduced risk of influenza infection in different human patient cohorts [37]. In contrast to mice and swine, IL2 gene expression was more strongly induced in infected macaques on both days. This may indicate macaques mount enhanced Th1 cell responses during CA04 virus infection compared to mice and swine (Figure 6, lower panel). As shown in Figure 6, we also found increased, expression of VDR/RXR Activation associated genes such as CYP24A1, encoding a hydroxylase involved in vitamin D catabolism, RXRG, and HR, a transcriptional corepressor of vitamin D receptor. Intriguingly, antimicrobial peptide, CAMP (also known as LL-37), was found to be downregulated in mouse and macaques infected with CA04 virus, and the decreased expression of CAMP in CA04 virus-infected macaques may be explained in part by the increased expression of APOA1 (Figure 6, top panel; Additional file 7: Table S4), which has been shown to bind and inhibit CAMP [38]. While there are several genes associated with VDR/RXR Activation that are differentially expressed in macaques infected with CA04 virus, vitamin D control of innate immune responses does not appear to play a major role during infection.

## Conclusions

We have shown differences in transcriptional responses to a single 2009 pandemic H1N1 influenza virus in three independent animal models. Our goal in performing a cross-species transcriptomic comparison was to identify shared and differential gene expression patterns to better evaluate the acute phase in different hosts. We found significant differences in expression of inflammatory response and lipid metabolism genes, which were likely impacted by glucocorticoid receptor and retinoid X receptor signaling complexes, such as LXR/RXR that is known to regulate cholesterol homeostasis during inflammation. The timing and magnitude of the host response is critical in determining disease outcome [39] and the success of immunomodulatory therapy (reviewed in [9]). For example, corticosteroid therapy in patients infected with pH1N1 virus was found to increase the risk of developing severe disease [40]. Metagenomic studies using clinical samples will be necessary to further understand the host response to pH1N1 virus and other etiological agents within the human population, as recently explored by Greninger et al. using a pan-viral microarray and deep sequencing to characterize pH1N1 virus from human nasopharyngeal aspirates [41]. Despite similar clinical outcomes, differences in the host transcriptional response could suggest that therapeutic targets identified in one host may not be relevant in another.

The gene expression differences elicited by CA04 virus within these three independent animal models demonstrate the disparate nature of the host response. There are particular advantages and disadvantages in modeling disease and immune responses to influenza infection in mice, macaques and swine. Mice, though largely resistant to infection with most human virus isolates, provide a tractable system for genetic manipulation to study key genes in the regulation of host responses to influenza virus. Non human primates are thought to more closely model the human response to influenza virus given their genetic and physiological similarities, while swine serve as a natural reservoir for influenza A viruses and have been linked to the emergence of some of the most notable influenza pandemics in recent history, including the H1N1 pandemic in 2009. There has been interest in determining the extent to which influenza viruses are able to cause disease in swine, particularly with regard to 1918 pandemic influenza virus that does not cause severe disease in swine like it does in mice and macaques [42]. This makes swine a unique model for future systems biology analyses that may help to uncover host responses contributing toward the emergence or maintenance of novel influenza viruses with pandemic potential.

## Methods

### Virus

2009 pandemic H1N1 influenza virus A/California/04/2009 (CA04) was isolated from a nasal swab of a <18 y.o. boy from San Diego, California [43].

### Animal models

Female BALB/c mice (*Mus musculus*), 6 to 8-week-old, were intranasally inoculated with 10^6^ plaque-forming units of CA04 virus in 50 μl (*n* = 9) or inoculated with 50 μl of phosphate-buffered saline (control; *n* = 8). Whole lungs were harvested from infected animals at days 1, 3 and 5 post-inoculation (*n* = 3 per time point) and from time-matched control animals (*n* = 3 on days 1 and 3 and *n* = 2 on day 5) for extraction of total RNA as previously described [10]. Crossbred pigs (*Sus Scrofa)*, 4-week-old, were inoculated intratracheally with either 10^6^ TCID_50_/pig egg-derived CA04 virus (*n* = 15) or mock inoculated with non-infectious cell culture supernatant (control; *n* = 15) as described elsewhere [44]. Animals were euthanized on 3, 5, and 7 dpi (*n* = 5 per time point). Cynomolgus macaques (*Macaca fascicularis*), 4 to 15 y.o., weighing 3.0-8.7 kg, were infected with CA04 virus (*n* = 6) under anesthesia through a combination of intratracheal (4 ml), intranasal (0.5 ml per nostril), conjunctival (0.5 ml per eyelid) and oral (1 ml) routes with a suspension containing 1×10^6^ TCID_50_/ml (total infectious dose was 7×10^6^ TCID_50_) as described elsewhere [7]. Animals were euthanized on 1 and 6 dpi (*n* = 2 per time point).

### Animal ethics

Mouse infection experiments were completed at the CDC under the guidance of the CDC’s Institutional Animal Care and Use Committee in an Association for Assessment and Accreditation of Laboratory Animal Care International-(AAALAC)-accredited animal facility. Swine infection experiments were completed at the Central States Research Center (CSRC), Inc BSL-3 facility (Oakland, NE) in compliance with the CSRC’s Institutional Animal Care and Use Committee studies. Macaque infection experiments were approved by the RML Institutional Animal Care and Use Committee (IACUC), and performed following the guidelines of AAALAC by certified staff in an AALAC approved facility.

### Real-time qRT-PCR

Real-time PCR was performed using a Custom TaqMan Gene Expression Assay (Applied Biosystems) designed for CA04 HA sequence (forward primer: AGCTCAGTGTCATCATTTGAAAGGT; reverse primer: GGACATGCTGCCGTTACAC; reporter: TTGGGCCATGAACTTG). cDNAs were generated using a QuantiTect reverse transcription kit (Qiagen). Samples from individual animals were run in quadruplicate. rRNA (18S) was used to normalize quantification of the target and quantification of normalized target was performed using the 2^-ΔΔCt^ calculation [45]. HA expression was quantified relative to expression of an endogenous control for each specie sample set that did not change with infection and expression in an uninfected lung sample. The following TaqMan Gene Expression Assays (Applied Biosystems) were used: Mfap1a (Assay ID Mm00849648_gH) served as the endogenous control for mouse samples, B2M (Assay ID Rh02847368_m1) served as the endogenous control for macaque samples, and RPS6 (Assay ID Ss03374061_g1) served as the endogenous control for swine samples. Average log10RQ expression is shown for each species at each time point ± standard deviation.

### Microarray hybridization

Total RNA isolated from lung tissue from individual animals on each day of euthanasia was used for oligonucleotide array experiments. For swine and mice, RNA isolated from mock-infected animals at each time point served as an uninfected reference. For cynomolgus macaques, a pool of RNA from the lungs of eight uninfected animals matched for age and sex was used as the uninfected reference. NanoDrop ND-1000 and Agilent 2100 Bioanalyzer instrumentation was used to determine the concentration and quality of all RNA samples. A total of 1500 ng Cy3-labeled probe was used for microarray slide hybridizations, thereby normalizing for the input RNA amount. Mouse, macaque and swine samples were measured with 4×44K commercial arrays from Agilent Technologies designed for each species, Mouse Whole Genome Gene Expression Microarray (G4122F), Rhesus Macaque Gene Expression Microarray (G2519F; Design ID: V2: 026806), and Porcine Gene Expression Microarray V1 (G2519F; Design ID: V1: 020109).

### Data normalization and accessibility

The background corrected data from the Feature Extraction output were normalized across replicates within each species using central tendency normalization (75% percentile, target 1000) within Genedata (Analyst 7.0). All primary microarray data have been deposited in NCBI's Gene Expression Omnibus (GEO) under GEO Series (GSE) accession number GSE40092. The primary microarray data are also available at the University of Washington’s Public Microarray Data Download site ( http://expression.microslu.washington.edu).

### Statistical analysis of microarray data

For mice and swine, Student’s t-test was performed on background corrected, normalized log-intensity data comparing CA04 virus-infected lung gene expression to time- and species-matched mock-infected lung gene expression at each time point (Unadjusted *P*-value < 0.05). For cynomologus macaques, Student’s t-test was performed on background corrected, normalized log-intensity data comparing CA04 virus-infected lung gene expression to an uninfected lung reference pool (*n* = 8) matched for age and sex at each time point (Unadjusted *P*-value < 0.05). Differentially expressed genes were then filtered to include only genes that changed at least two-fold compared to mock on at least one day within each species. Statistical comparison using Student’s t-test with the Benjamini-Hochberg multiple testing correction [46] (Adjusted *P*-value < 0.05) and no fold-change parameter resulted in very few macaque genes that were statistically significant, though there was considerable overlap in the DE gene sets identified from the two tests for each species.

### Functional analysis of differential gene expression data

Functional analysis of DE genes was performed using Ingenuity Pathways Analysis (Ingenuity Systems), which analyzes the experimental dataset in the context of known biological response and regulatory networks in the Ingenuity Pathways Knowledge Base (IPKB). Ensembl human gene annotations were used for functional analyses of macaque and swine gene sets (Additional file 8: Table S5). The right-tailed Fisher’s Exact test was used to determine the statistical significance of each biological function assigned to the gene expression data, and the Benjamini-Hochberg (B-H) Multiple Testing Correction was applied to p-values to reduce the likelihood that statistical associations were due to random chance.

### Transcription factor enrichment analysis

Upstream Regulator Analysis in IPA incorporates expression of downstream target genes from the experimental dataset and compiled knowledge of reported relationships between regulators and their known target genes within IPKB. This analytical tool was used to predict upstream regulators and infer their activation state by calculating a z-score that determines whether gene expression changes for known targets of each regulator (z > 2, regulator predicted as “activated” and z < -2, regulator predicted as “inhibited”. Transcription factor binding motif enrichment was performed with PSCAN [47] using Position Weight Matrices (PWM) for human and mouse species obtained from JASPAR CORE database [48] and the promoter of each target gene defined from -450 to +50 nucleotides relative to the TSS. PSCAN computes a *z*-test P-value for each regulator, which is an assessment of whether there is significant representation (P < 0.001) of the regulator DNA-binding motif in promoters of the queried genes.

### Transcription factor DNA-binding promoter analysis

Transcription factor (TF) DNA-binding analysis was performed using STAT1 (human MA0137.2) and HNF1A (mouse MA153.1) PWMs obtained from the JASPAR CORE database [48] and STAT1 (mouse M00224) and HNF1A (human M00206) PWMs obtained from the TRANSFAC database 7.0 [49]. Promoters sequences of genes analyzed were defined from -450 to +50 nucleotides relative to the TSS and were retrieved from the Ensembl database (release 66). *Macaca mulatta* sequences were used instead of *Macaca fascicularis* sequences for the TF DNA-binding analysis*.* P-values associated for each target gene were calculated using the TFM-Pvalue analytical tool described in [50] and distribution of nucleotides amongst the promoter sequences have been taken into account for background correction. Significant target genes have been identified as having a P-value < 10^-4^.

## Endnotes

^a^Safronetz and Feldmann, personal communications Laboratory of Virology, Division of Intramural Research, National Institute of Allergy and Infectious Diseases, National Institutes of Health, Rocky Mountain Laboratories, Hamilton, MT 59840, USA.

## Abbreviations

Mm: *Mus musculus*; Mf: *Macaca fascicularis*; Ss: *Sus scrofa*; DE: Differentially expressed; FC: Fold change; IPA: Ingenuity Pathways Analysis; IPKDB: Ingenuity Pathway Knowledge Database; TF: Transcription factor; RXR: Retinoid X receptor; LXR: Liver-X-receptor; VDR: Vitamin D receptor; pH1N1: 2009 pandemic H1N1 influenza virus; CA04: Influenza A/California/04/2009 virus; IFN: Interferon; TSS: Transcription Start Site; PWM: Position Weight Matrix.

## Competing interests

The authors declare that they have no competing interests.

## Authors’ contributions

Authors TMT carried out the mouse infection experiment. WM and JAR carried out the swine infection experiment. DS and HF carried out the macaque infection experiment. SB, JTG and MGK participated in the design of the microarray experiment. SB performed the statistical analysis and helped to draft the manuscript. NT performed the transcription factor analysis. JTG performed the functional analysis and wrote the manuscript. All authors read and approved the final manuscript.

## Supplementary Material

Additional file 1 **Figure S1.** Relative influenza HA gene expression in the lung of infected mice, macaques and swine. CA04 virus HA mRNA was quantified in each lung sample relative to gene expression of an endogenous control that did not change with infection (Mfap1a for mouse; B2M for macaque; Ss03374061_g1 for swine) and expression in an infected lung sample that did not have detectable CA04 HA expression. Average log10RQ expression ± SD is shown for each species at each time point (mice, *n* =3 per time point; macaques, *n* = 2 per time point; swine, *n* = 3 per time point). (PDF 288 kb) Click here for file

Additional file 2 **Table S1.** Summary of the top 10 Canonical Pathways enriched in mouse, macaque and swine CA04 infection models including the differentially expressed genes associated with each pathway. (XLSX 15 kb) Click here for file

Additional file 3 **Table S2.** Functional analysis of DE genes from mice, macaques and swine infected with CA04 virus. (DOC 77 kb) Click here for file

Additional file 4 **Table S3.** Gene expression of 53 DE genes commonly differentially expressed in mouse, macaque and swine lung infected with CA04 virus. (DOC 77 kb) Click here for file

Additional file 5 **Figure S2.** Pathway enrichment in mouse, macaque and swine infected lung unique to each species. Ingenuity Pathway Analysis was used to determine the top 5 Canonical Pathways. Fisher’s Exact test *p*-value was used to rank the significance associated for each Canonical Pathway. (PDF 383 kb) Click here for file

Additional file 6 **Figure S3.** Kinetics of STAT1 and HNF1A target gene expression in mouse, macaque and swine infected with CA04 virus. A) Transcription factor DNA-binding analysis used matrices obtained from JASPAR CORE and TRANSFAC databases. Human matrices were applied to *Macaca mulatta* and *Sus Scrofa* genome sequence scans. For STAT1, human MA0137.2 matrix and mouse M00224 matrix were used. For HNF1A, human M00206 matrix and mouse MA153.1 matrix were used. B) Average fold change gene expression compared to mock for STAT1 and HNF1A target genes identified by genome scans with matrices in part A. DE genes are shaded gray and target genes of STAT1 (left panel) and HNF1A (right panel) are shaded purple. The STAT1 DE gene is highlighted in red. C) Average log_10_(ratio) expression of STAT1 (left panel) and HNF1A (right panel) target genes identified using IPA Upstream Regulator Analysis. In mice and swine, infected lung gene expression is referenced to specie-matched mock at each time point. In macaques, infected lung gene expression is relative to an uninfected lung reference pool at each time point. Red indicates expression was increased relative to the control reference and green indicates that expression was decreased relative to the control reference. Saturation is 4-fold. (PDF 619 kb) Click here for file

Additional file 7 **Table S4.** Gene expression of LXR/RXR and VDR/RXR genes in mouse, macaque and swine lung infected with CA04 virus. (DOC 69 kb) Click here for file

Additional file 8 **Table S5.** Summary of CA04 induced DE gene sets for each species. (DOC 30 kb) Click here for file

## References

[B1] Smith GJ Vijaykrishna D Bahl J Lycett SJ Worobey M Pybus OG Ma SK Cheung CL Raghwani J Bhatt S Origins and evolutionary genomics of the 2009 swine-origin H1N1 influenza A epidemic Nature 2009 459 7250 1122 1125 10.1038/nature08182 19516283

[B2] Chowell G Bertozzi SM Colchero MA Lopez-Gatell H Alpuche-Aranda C Hernandez M Miller MA Severe respiratory disease concurrent with the circulation of H1N1 influenza N Engl J Med 2009 361 7 674 679 10.1056/NEJMoa0904023 19564633

[B3] Perez-Padilla R De la Rosa-Zamboni D Ponce De Leon S Hernandez M Quinones-Falconi F Bautista E Ramirez-Venegas A Rojas-Serrano J Ormsby CE Corrales A Pneumonia and respiratory failure from swine-origin influenza A (H1N1) in Mexico N Engl J Med 2009 361 7 680 689 10.1056/NEJMoa0904252 19564631

[B4] Dawood FS Iuliano AD Reed C Meltzer MI Shay DK Cheng PY Bandaranayake D Breiman RF Brooks WA Buchy P Estimated global mortality associated with the first 12 months of 2009 pandemic influenza A H1N1 virus circulation: a modelling study Lancet Infect Dis 2012 12 9 687 695 10.1016/S1473-3099(12)70121-4 22738893

[B5] de Jong MD Simmons CP Thanh TT Hien VM Smith GJ Chau TN Hoang DM Chau NV Khanh TH Dong VC Fatal outcome of human influenza A (H5N1) is associated with high viral load and hypercytokinemia Nat Med 2006 12 10 1203 1207 10.1038/nm1477 16964257 PMC4333202

[B6] Kobasa D Jones SM Shinya K Kash JC Copps J Ebihara H Hatta Y Kim JH Halfmann P Hatta M Aberrant innate immune response in lethal infection of macaques with the 1918 influenza virus Nature 2007 445 7125 319 323 10.1038/nature05495 17230189

[B7] Safronetz D Rockx B Feldmann F Belisle SE Palermo RE Brining D Gardner D Proll SC Marzi A Tsuda Y Pandemic swine-origin H1N1 influenza A virus isolates show heterogeneous virulence in macaques J Virol 2011 85 3 1214 1223 10.1128/JVI.01848-10 21084481 PMC3020514

[B8] Itoh Y Shinya K Kiso M Watanabe T Sakoda Y Hatta M Muramoto Y Tamura D Sakai-Tagawa Y Noda T In vitro and in vivo characterization of new swine-origin H1N1 influenza viruses Nature 2009 460 7258 1021 1025 19672242 10.1038/nature08260 PMC2748827

[B9] Tisoncik JR Korth MJ Simmons CP Farrar J Martin TR Katze MG Into the eye of the cytokine storm Microbiol Mol Biol Rev 2012 76 1 16 32 10.1128/MMBR.05015-11 22390970 PMC3294426

[B10] Josset L Belser JA Pantin-Jackwood MJ Chang JH Chang ST Belisle SE Tumpey TM Katze MG Implication of inflammatory macrophages, nuclear receptors and interferon regulatory factors in increased virulence of pandemic 2009 H1N1 influenza A virus after host adaptation J Virol 2012 86 13 7192 7206 10.1128/JVI.00563-12 22532695 PMC3416346

[B11] Ozawa M Basnet S Burley LM Neumann G Hatta M Kawaoka Y Impact of amino acid mutations in PB2, PB1-F2, and NS1 on the replication and pathogenicity of pandemic (H1N1) 2009 influenza viruses J Virol 2011 85 9 4596 4601 10.1128/JVI.00029-11 21325408 PMC3126221

[B12] Hai R Schmolke M Varga ZT Manicassamy B Wang TT Belser JA Pearce MB Garcia-Sastre A Tumpey TM Palese P PB1-F2 expression by the 2009 pandemic H1N1 influenza virus has minimal impact on virulence in animal models J Virol 2010 84 9 4442 4450 10.1128/JVI.02717-09 20181699 PMC2863736

[B13] Hale BG Steel J Medina RA Manicassamy B Ye J Hickman D Hai R Schmolke M Lowen AC Perez DR Inefficient control of host gene expression by the 2009 pandemic H1N1 influenza A virus NS1 protein J Virol 2010 84 14 6909 6922 10.1128/JVI.00081-10 20444891 PMC2898253

[B14] Herfst S van den Brand JM Schrauwen EJ de Wit E Munster VJ van Amerongen G Linster M Zaaraoui F van Ijcken WF Rimmelzwaan GF Pandemic 2009 H1N1 influenza virus causes diffuse alveolar damage in cynomolgus macaques Vet Pathol 2010 47 6 1040 1047 10.1177/0300985810374836 20647595

[B15] Ma W Belisle SE Mosier D Li X Stigger-Rosser E Liu Q Qiao C Elder J Webby R Katze MG 2009 pandemic H1N1 influenza virus causes disease and upregulation of genes related to inflammatory and immune responses, cell death, and lipid metabolism in pigs J Virol 2011 85 22 11626 11637 10.1128/JVI.05705-11 21900171 PMC3209293

[B16] Ma W Liu Q Bawa B Qiao C Qi W Shen H Chen Y Ma J Li X Webby RJ The neuraminidase and matrix genes of the 2009 pandemic influenza H1N1 virus cooperate functionally to facilitate efficient replication and transmissibility in pigs J Gen Virol 2012 93 Pt 6 1261 1268 22337640 10.1099/vir.0.040535-0 PMC3755515

[B17] Lakdawala SS Lamirande EW Suguitan AL Jr Wang W Santos CP Vogel L Matsuoka Y Lindsley WG Jin H Subbarao K Eurasian-origin gene segments contribute to the transmissibility, aerosol release, and morphology of the 2009 pandemic H1N1 influenza virus PLoS Pathog 2011 7 12 e1002443 10.1371/journal.ppat.1002443 22241979 PMC3248560

[B18] Chou YY Albrecht RA Pica N Lowen AC Richt JA Garcia-Sastre A Palese P Hai R The M segment of the 2009 new pandemic H1N1 influenza virus is critical for its high transmission efficiency in the guinea pig model J Virol 2011 85 21 11235 11241 10.1128/JVI.05794-11 21880744 PMC3194962

[B19] Lee SM Chan RW Gardy JL Lo CK Sihoe AD Kang SS Cheung TK Guan YI Chan MC Hancock RE Systems-level comparison of host responses induced by pandemic and seasonal influenza A H1N1 viruses in primary human type I-like alveolar epithelial cells in vitro Respir Res 2010 11 147 10.1186/1465-9921-11-147 21029402 PMC2988725

[B20] McDermott JE Shankaran H Eisfeld AJ Belisle SE Neuman G Li C McWeeney S Sabourin C Kawaoka Y Katze MG Conserved host response to highly pathogenic avian influenza virus infection in human cell culture, mouse and macaque model systems BMC Syst Biol 2011 5 190 10.1186/1752-0509-5-190 22074594 PMC3229612

[B21] Perrone LA Plowden JK Garcia-Sastre A Katz JM Tumpey TM H5N1 and 1918 pandemic influenza virus infection results in early and excessive infiltration of macrophages and neutrophils in the lungs of mice PLoS Pathog 2008 4 8 e1000115 10.1371/journal.ppat.1000115 18670648 PMC2483250

[B22] Marcelin G Aldridge JR Duan S Ghoneim HE Rehg J Marjuki H Boon AC McCullers JA Webby RJ Fatal outcome of pandemic H1N1 2009 influenza virus infection is associated with immunopathology and impaired lung repair, not enhanced viral burden, in pregnant mice J Virol 2011 85 21 11208 11219 10.1128/JVI.00654-11 21865394 PMC3194964

[B23] Gatzka M Piekorz R Moriggl R Rawlings J Ihle JN A role for STAT5A/B in protection of peripheral T-lymphocytes from postactivation apoptosis: insights from gene expression profiling Cytokine 2006 34 3–4 143 154 16757175 10.1016/j.cyto.2006.04.003

[B24] Chen CH Zhang XQ Lo CW Liu PF Liu YT Gallo RL Hsieh MF Schooley RT Huang CM The essentiality of alpha-2-macroglobulin in human salivary innate immunity against new H1N1 swine origin influenza A virus Proteomics 2010 10 12 2396 2401 10.1002/pmic.200900775 20391540 PMC2890046

[B25] Escoubet-Lozach L Benner C Kaikkonen MU Lozach J Heinz S Spann NJ Crotti A Stender J Ghisletti S Reichart D Mechanisms establishing TLR4-responsive activation states of inflammatory response genes PLoS Genet 2011 7 12 e1002401 10.1371/journal.pgen.1002401 22174696 PMC3234212

[B26] John S Sabo PJ Thurman RE Sung MH Biddie SC Johnson TA Hager GL Stamatoyannopoulos JA Chromatin accessibility pre-determines glucocorticoid receptor binding patterns Nat Genet 2011 43 3 264 268 10.1038/ng.759 21258342 PMC6386452

[B27] Zelcer N Tontonoz P Liver X receptors as integrators of metabolic and inflammatory signaling J Clin Invest 2006 116 3 607 614 10.1172/JCI27883 16511593 PMC1386115

[B28] Beard JA Bearden A Striker R Vitamin D and the anti-viral state J Clin Virol 2011 50 3 194 200 10.1016/j.jcv.2010.12.006 21242105 PMC3308600

[B29] Anand PK Kaul D Sharma M Synergistic action of vitamin D and retinoic acid restricts invasion of macrophages by pathogenic mycobacteria J Microbiol Immunol Infect 2008 41 1 17 25 18327422

[B30] Joseph SB Bradley MN Castrillo A Bruhn KW Mak PA Pei L Hogenesch J O'Connell RM Cheng G Saez E LXR-dependent gene expression is important for macrophage survival and the innate immune response Cell 2004 119 2 299 309 10.1016/j.cell.2004.09.032 15479645

[B31] Castrillo A Joseph SB Vaidya SA Haberland M Fogelman AM Cheng G Tontonoz P Crosstalk between LXR and toll-like receptor signaling mediates bacterial and viral antagonism of cholesterol metabolism Mol Cell 2003 12 4 805 816 10.1016/S1097-2765(03)00384-8 14580333

[B32] Galkina E Ley K Immune and inflammatory mechanisms of atherosclerosis (*) Annu Rev Immunol 2009 27 165 197 10.1146/annurev.immunol.021908.132620 19302038 PMC2734407

[B33] Kalayoglu MV Byrne GI Induction of macrophage foam cell formation by Chlamydia pneumoniae J Infect Dis 1998 177 3 725 729 10.1086/514241 9498454

[B34] Portugal LR Fernandes LR Pietra Pedroso VS Santiago HC Gazzinelli RT Alvarez-Leite JI Influence of low-density lipoprotein (LDL) receptor on lipid composition, inflammation and parasitism during Toxoplasma gondii infection Microbes Infect 2008 10 3 276 284 10.1016/j.micinf.2007.12.001 18316222

[B35] Liu PT Stenger S Li H Wenzel L Tan BH Krutzik SR Ochoa MT Schauber J Wu K Meinken C Toll-like receptor triggering of a vitamin D-mediated human antimicrobial response Science 2006 311 5768 1770 1773 10.1126/science.1123933 16497887

[B36] Hansdottir S Monick MM Hinde SL Lovan N Look DC Hunninghake GW Respiratory epithelial cells convert inactive vitamin D to its active form: potential effects on host defense J Immunol 2008 181 10 7090 7099 18981129 10.4049/jimmunol.181.10.7090 PMC2596683

[B37] Urashima M Segawa T Okazaki M Kurihara M Wada Y Ida H Randomized trial of vitamin D supplementation to prevent seasonal influenza A in schoolchildren Am J Clin Nutr 2010 91 5 1255 1260 10.3945/ajcn.2009.29094 20219962

[B38] Wang Y Agerberth B Lothgren A Almstedt A Johansson J Apolipoprotein A-I binds and inhibits the human antibacterial/cytotoxic peptide LL-37 J Biol Chem 1998 273 50 33115 33118 10.1074/jbc.273.50.33115 9837875

[B39] Chang ST Tchitchek N Ghosh D Benecke A Katze MG A chemokine gene expression signature derived from meta-analysis predicts the pathogenicity of viral respiratory infections BMC Syst Biol 2011 5 202 10.1186/1752-0509-5-202 22189154 PMC3297540

[B40] Diaz E Martin-Loeches I Canadell L Vidaur L Suarez D Socias L Estella A Gil Rueda B Guerrero JE Valverdu-Vidal M Corticosteroid therapy in patients with primary viral pneumonia due to pandemic (H1N1) 2009 influenza J Infect 2012 64 3 311 318 10.1016/j.jinf.2011.12.010 22240033

[B41] Greninger AL Chen EC Sittler T Scheinerman A Roubinian N Yu G Kim E Pillai DR Guyard C Mazzulli T A metagenomic analysis of pandemic influenza A (2009 H1N1) infection in patients from North America PLoS One 2010 5 10 e13381 10.1371/journal.pone.0013381 20976137 PMC2956640

[B42] Weingartl HM Albrecht RA Lager KM Babiuk S Marszal P Neufeld J Embury-Hyatt C Lekcharoensuk P Tumpey TM Garcia-Sastre A Experimental infection of pigs with the human 1918 pandemic influenza virus J Virol 2009 83 9 4287 4296 10.1128/JVI.02399-08 19224986 PMC2668479

[B43] Centers for Disease Control and Prevention Swine influenza A (H1N1) infection in two children--Southern California, March-April 2009 MMWR Morb Mortal Wkly Rep 2009 58 15 400 402 19390508

[B44] Richt JA Lager KM Janke BH Woods RD Webster RG Webby RJ Pathogenic and antigenic properties of phylogenetically distinct reassortant H3N2 swine influenza viruses cocirculating in the United States J Clin Microbiol 2003 41 7 3198 3205 10.1128/JCM.41.7.3198-3205.2003 12843064 PMC165376

[B45] Livak KJ Schmittgen TD Analysis of relative gene expression data using real-time quantitative PCR and the 2(-Delta Delta C(T)) Method Methods 2001 25 4 402 408 10.1006/meth.2001.1262 11846609

[B46] Reiner A Yekutieli D Benjamini Y Identifying differentially expressed genes using false discovery rate controlling procedures Bioinformatics 2003 19 3 368 375 10.1093/bioinformatics/btf877 12584122

[B47] Zambelli F Pesole G Pavesi G Pscan: finding over-represented transcription factor binding site motifs in sequences from co-regulated or co-expressed genes Nucleic Acids Res 2009 37 Web Server issue W247 252 19487240 10.1093/nar/gkp464 PMC2703934

[B48] Sandelin A Alkema W Engstrom P Wasserman WW Lenhard B JASPAR: an open-access database for eukaryotic transcription factor binding profiles Nucleic Acids Res 2004 32 Database issue D91 94 14681366 10.1093/nar/gkh012 PMC308747

[B49] Matys V Fricke E Geffers R Gossling E Haubrock M Hehl R Hornischer K Karas D Kel AE Kel-Margoulis OV TRANSFAC: transcriptional regulation, from patterns to profiles Nucleic Acids Res 2003 31 1 374 378 10.1093/nar/gkg108 12520026 PMC165555

[B50] Touzet H Varre JS Efficient and accurate P-value computation for Position Weight Matrices Algorithms Mol Biol 2007 2 15 10.1186/1748-7188-2-15 18072973 PMC2238751

