# Supplementary figures and images for: 2009 pandemic H1N1 influenza virus elicits similar clinical course but differential host transcriptional response in mouse, macaque, and swine infection models
Source: BMC Genomics. 2012 Nov 15;13:627. doi: 10.1186/1471-2164-13-627 (PMC3532173; doi:10.1186/1471-2164-13-627)

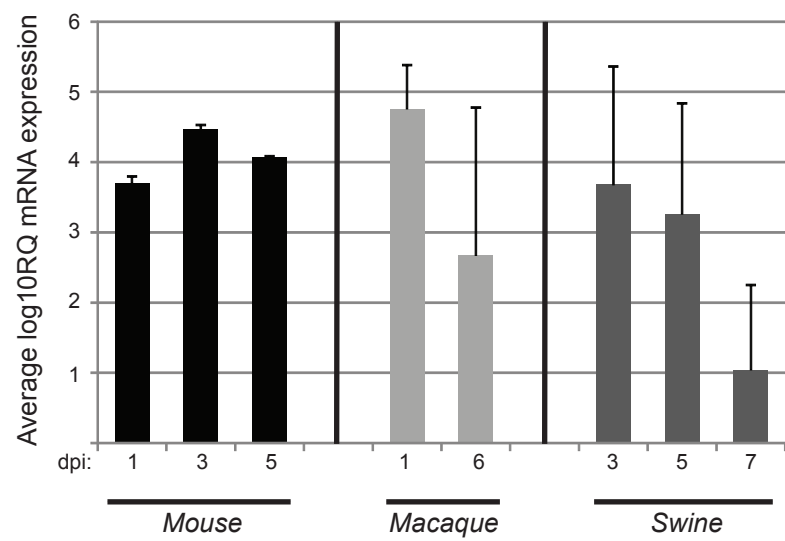

Supplement: Additional file 1 — Figure S1. Relative influenza HA gene expression in the lung of infected mice, macaques and swine. CA04 virus HA mRNA was quantified in each lung sample relative to gene expression of an endogenous control that did not change with infection (Mfap1a for mouse; B2M for macaque; Ss03374061_g1 for swine) and expression in an infected lung sample that did not have detectable CA04 HA expression. Average log10RQ expression ± SD is shown for each species at each time point (mice, n =3 per time point; macaques, n = 2 per time point; swine, n = 3 per time point). (PDF 288 kb) [file 1471-2164-13-627-S1.pdf]

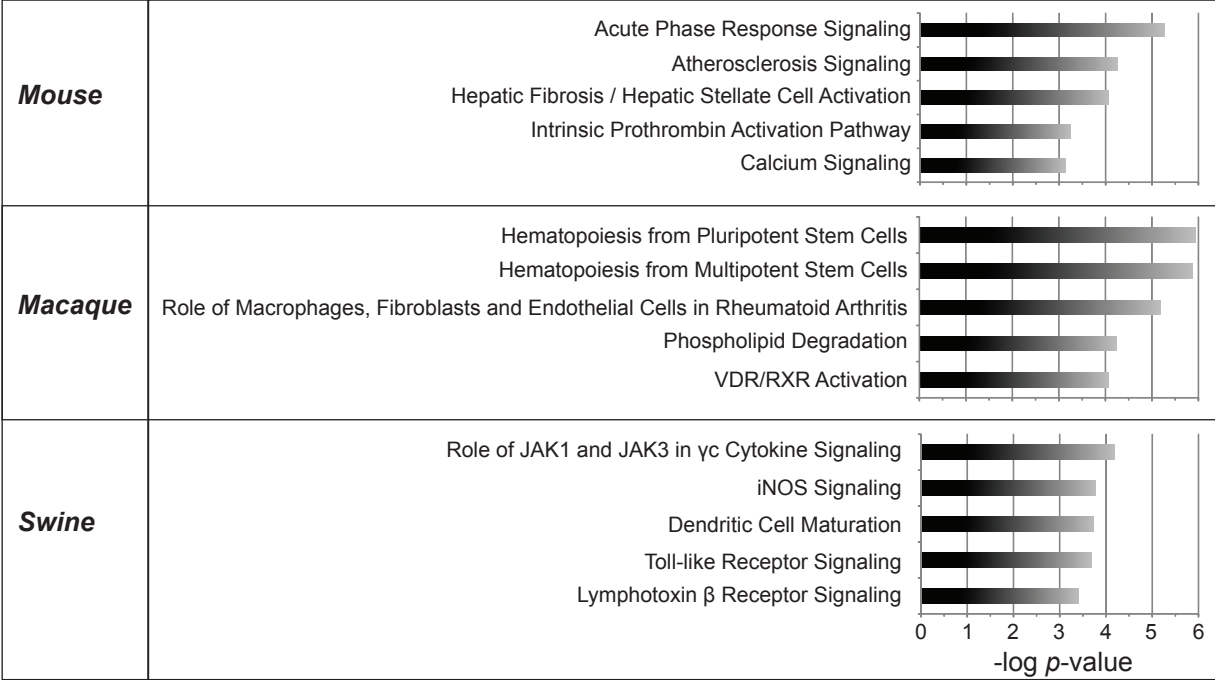

Supplement: Additional file 5 — Figure S2. Pathway enrichment in mouse, macaque and swine infected lung unique to each species. Ingenuity Pathway Analysis was used to determine the top 5 Canonical Pathways. Fisher’s Exact test p-value was used to rank the significance associated for each Canonical Pathway. (PDF 383 kb) [file 1471-2164-13-627-S5.pdf]

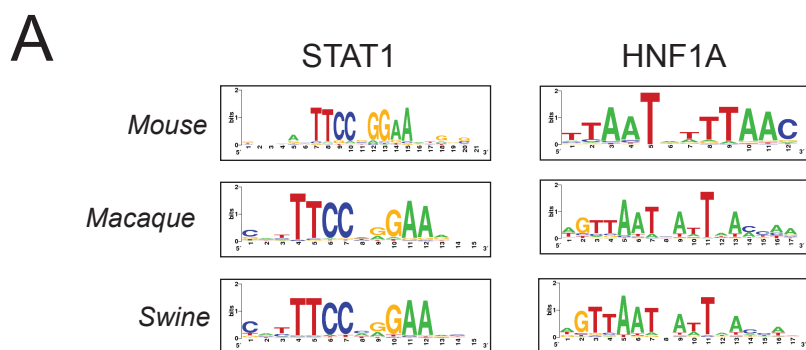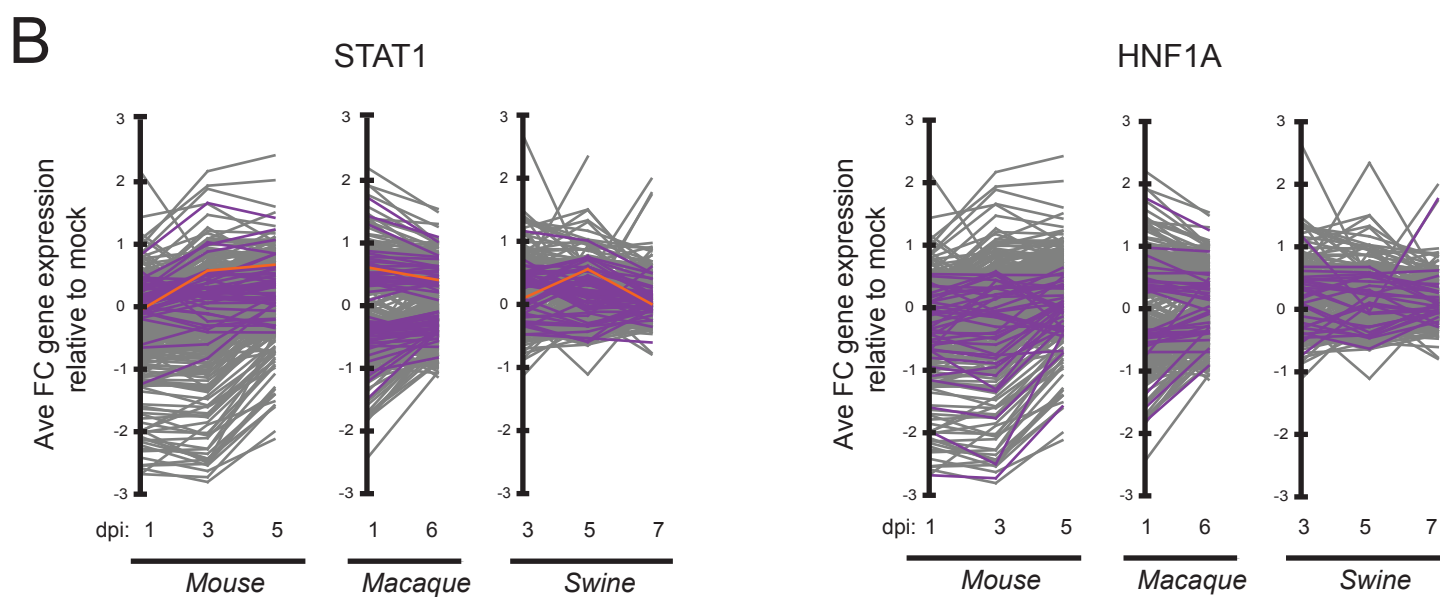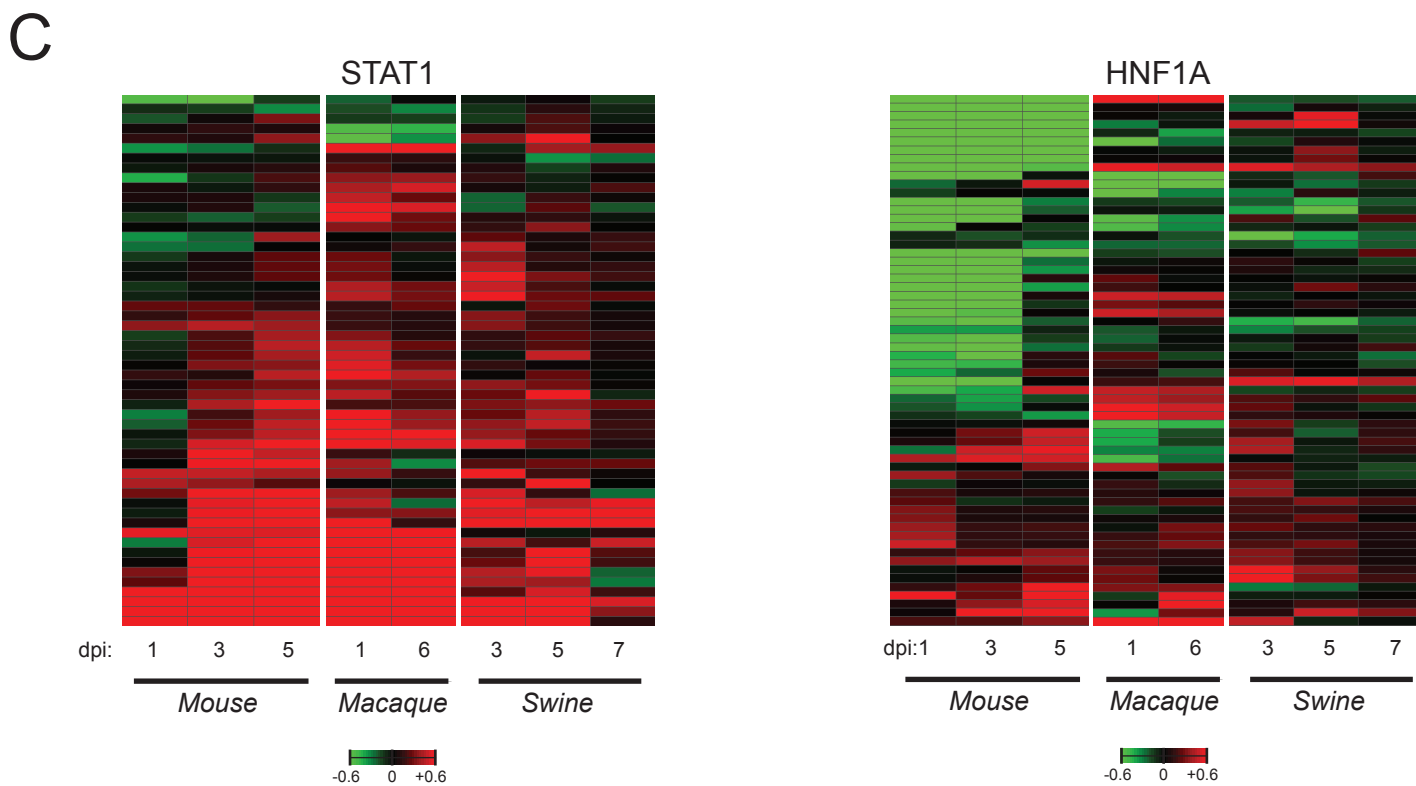

Supplement: Additional file 6 — Figure S3. Kinetics of STAT1 and HNF1A target gene expression in mouse, macaque and swine infected with CA04 virus. A) Transcription factor DNA-binding analysis used matrices obtained from JASPAR CORE and TRANSFAC databases. Human matrices were applied to Macaca mulatta and Sus Scrofa genome sequence scans. For STAT1, human MA0137.2 matrix and mouse M00224 matrix were used. For HNF1A, human M00206 matrix and mouse MA153.1 matrix were used. B) Average fold change gene expression compared to mock for STAT1 and HNF1A target genes identified by genome scans with matrices in part A. DE genes are shaded gray and target genes of STAT1 (left panel) and HNF1A (right panel) are shaded purple. The STAT1 DE gene is highlighted in red. C) Average log10(ratio) expression of STAT1 (left panel) and HNF1A (right panel) target genes identified using IPA Upstream Regulator Analysis. In mice and swine, infected lung gene expression is referenced to specie-matched mock at each time point. In macaques, infected lung gene expression is relative to an uninfected lung reference pool at each time point. Red indicates expression was increased relative to the control reference and green indicates that expression was decreased relative to the control reference. Saturation is 4-fold. (PDF 619 kb) [file 1471-2164-13-627-S6.pdf]
