# Supplementary material for: 2009 pandemic H1N1 influenza virus elicits similar clinical course but differential host transcriptional response in mouse, macaque, and swine infection models
Source: BMC Genomics. 2012 Nov 15;13:627. doi: 10.1186/1471-2164-13-627 (PMC3532173; doi:10.1186/1471-2164-13-627)
Supplement: Additional file 3 — Table S2. Functional analysis of DE genes from mice, macaques and swine infected with CA04 virus. (DOC 77 kb) [file 1471-2164-13-627-S3.doc]

| **Supplementary Table 2.** Functional analysis of DE genes from mice, macaques and swine infected with CA04 virus | | | |
| --- | --- | --- | --- |
| **Animal model** | **Categories and Bio Functions** | ***p-*value range** | **No. Molecules** |
| ***Mouse*** | **Disease and Disorders** |  |  |
|  | Inflammatory Response | 1.05E-34 – 1.14E-06 | 175 |
|  | Organismal Injury and Abnormalities | 7.11E-22 – 1.16E-06 | 120 |
|  | Connective Tissue Disorders | 1.06E-19 – 9.61E-09 | 106 |
|  | Inflammatory Disease | 1.06E -19 – 1.16E-06 | 136 |
|  | Skeletal and Muscular Disorders | 1.06E -19 – 5.25E-08 | 148 |
|  | **Molecular and Cellular Functions** |  |  |
|  | Cell-To-Cell Signaling and Interaction | 7.28E-23 – 1.03E-06 | 149 |
|  | Cellular Function and Maintenance | 2.60E-19 – 1.07E-06 | 148 |
|  | Cellular Movement | 5.97E-18 – 1.14E-06 | 121 |
|  | Cell Death | 8.89E-18 – 1.12E-06 | 218 |
|  | Cellular Growth and Proliferation | 2.99E-17 – 6.61E-07 | 213 |
|  | **Physiological System Development and Function** |  |  |
|  | Tissue Morphology | 2.17E-22 – 6.34E-06 | 179 |
|  | Hematological System Development and Function | 7.64E-22 – 1.14E-06 | 175 |
|  | Immune Cell Trafficking | 1.10E-19 – 1.14E-06 | 118 |
|  | Tissue Development | 2.25E-16 – 8.60E-07 | 188 |
|  | Hematopoiesis | 2.19E-15 – 3.06E-07 | 92 |
|  |  | | |
| ***Macaque*** | **Disease and Disorders** |  |  |
|  | Inflammatory Response | 2.83E-21 – 1.57E-04 | 166 |
|  | Cancer | 1.40E-13 – 1.49E-04 | 278 |
|  | Infectious Disease | 3.70E-12 – 1.40E-04 | 125 |
|  | Renal and Urological Disease | 1.29E-09 – 1.98E-05 | 88 |
|  | Cardiovascular Disease | 7.33E-09 – 7.51E-05 | 95 |
|  | **Molecular and Cellular Functions** |  |  |
|  | Cell Death | 2.79E-18 – 1.61E-04 | 247 |
|  | Cell-To-Cell Signaling and Interaction | 1.28E-15 – 1.44E-04 | 162 |
|  | Cellular Function and Maintenance | 9.31E-15 – 1.50E-04 | 177 |
|  | Lipid Metabolism | 1.15E-13 – 1.50E-04 | 137 |
|  | Molecular Transport | 1.15E-13 – 1.50E-04 | 157 |
|  | **Physiological System Development and Function** |  |  |
|  | Tissue Morphology | 5.47E-16 – 1.60E-04 | 194 |
|  | Hematological System Development and Function | 1.28E-15 – 1.50E-04 | 169 |
|  | Organismal Survival | 1.14E-14 – 5.66E-09 | 151 |
|  | Immune Cell Trafficking | 5.21E-14 – 1.31E-04 | 117 |
|  | Endocrine System Development and Function | 5.91E-10 – 9.95E-05 | 53 |
|  |  |  |  |
| ***Swine*** | **Disease and Disorders** |  |  |
|  | Inflammatory Response | 1.56E-32 – 6.28E-05 | 160 |
|  | Cancer | 7.54E-18 – 6.02E-05 | 230 |
|  | Immunological Disease | 6.22E-17 – 6.39E-05 | 127 |
|  | Infectious Disease | 6.36E-17 – 6.28E-05 | 125 |
|  | Connective Tissue Disorders | 2.61E-16 – 6.32E-05 | 94 |
|  | **Molecular and Cellular Functions** |  |  |
|  | Cell-To-Cell Signaling and Interaction | 1.26E-25 – 6.76E-05 | 148 |
|  | Cellular Function and Maintenance | 4.47E-21 – 4.64E-05 | 130 |
|  | Cellular Growth and Proliferation | 2.50E-20 – 6.28E-05 | 206 |
|  | Cellular Movement | 1.19E-18 – 6.76E-05 | 127 |
|  | Cell Death | 7.04E-18 – 6.67E-05 | 196 |
|  | **Physiological System Development and Function** |  |  |
|  | Hematological System Development and Function | 1.26E-25 – 6.34E-05 | 165 |
|  | Immune Cell Trafficking | 4.60E-25 – 6.76E-05 | 109 |
|  | Tissue Morphology | 1.12E-23 – 6.28E-05 | 139 |
|  | Hematopoiesis | 4.47E-12 – 5.87E-05 | 87 |
|  | Organismal Survival | 1.46E-10 – 4.57E-08 | 118 |
|  |  |  |  |

Ingenuity Pathway Analysis was used to determine the top Bio Functions associated with the IPA Categories Disease and Disorders, Molecular and Cellular Functions, and Physiological System Development and Function. Fisher’s Exact test *p*-value was used to rank the significance associated for each Bio Function.
