# Supplementary material for: 2009 pandemic H1N1 influenza virus elicits similar clinical course but differential host transcriptional response in mouse, macaque, and swine infection models
Source: BMC Genomics. 2012 Nov 15;13:627. doi: 10.1186/1471-2164-13-627 (PMC3532173; doi:10.1186/1471-2164-13-627)
Supplement: Additional file 4 — Table S3. Gene expression of 53 DE genes commonly differentially expressed in mouse, macaque and swine lung infected with CA04 virus. (DOC 77 kb) [file 1471-2164-13-627-S4.doc]

| **Supplementary Table 3**. Gene expression of 53 DE genes commonly differentially expressed in mouse, macaque and swine lung infected with CA04 virus | | | | | | | | | |
| --- | --- | --- | --- | --- | --- | --- | --- | --- | --- |
| **Gene Symbol** | **Ensembl Gene ID** | ***Mouse*** | | | ***Macaque*** | | ***Swine*** | | |
|  |  | **1 dpi** | **3 dpi** | **5 dpi** | **1 dpi** | **6 dpi** | **3 dpi** | **5 dpi** | **7 dpi** |
|  |  |  |  |  |  |  |  |  |  |
| CLCA1 | ENSMUSG00000028255 | -0.74 | -0.41 | 0.60 | -1.70 | -0.49 | 0.89 | -0.25 | 0.83 |
| CYP1A1 | ENSMUSG00000032315 | 0.00 | -0.25 | -0.37 | -0.97 | -1.08 | 0.16 | 0.45 | 0.77 |
| GABRP | ENSMUSG00000020159 | -0.07 | 0.32 | 0.07 | -1.12 | -0.77 | 0.84 | 0.87 | 0.97 |
| **PLUNC** | **ENSMUSG00000027483** | **-0.53** | **-0.38** | **-0.43** | **-0.12** | **-0.81** | **1.15** | **0.02** | **1.75** |
| SLC27A2 | ENSMUSG00000027359 | -0.68 | -0.62 | 0.07 | -0.52 | -0.08 | -0.17 | -1.11 | 0.07 |
| ESM1 | ENSMUSG00000042379 | -0.22 | -0.06 | -0.50 | -0.33 | -0.48 | -0.51 | -0.25 | -0.30 |
| POLR1E | ENSMUSG00000028318 | 0.31 | 0.07 | 0.16 | -0.38 | -0.25 | 0.30 | 0.03 | -0.09 |
| **CD8A** | **ENSMUSG00000053977** | **-0.28** | **0.15** | **0.34** | **-0.25** | **-0.32** | **0.24** | **0.52** | **0.46** |
| EXO1 | ENSMUSG00000039748 | 0.23 | 0.47 | 0.65 | -0.87 | 0.18 | 0.22 | 0.43 | 0.11 |
| KIF20A | ENSMUSG00000003779 | 0.03 | 0.61 | 0.72 | -0.36 | 0.28 | 0.09 | 0.48 | 0.31 |
| TTN | ENSMUSG00000051747 | 0.25 | 0.36 | -0.07 | 0.07 | 0.01 | -0.23 | -0.24 | -0.12 |
| MYH4 | ENSMUSG00000057003 | 0.11 | 0.59 | -0.06 | 0.45 | 0.51 | -0.36 | -0.16 | -0.15 |
| NAGS | ENSMUSG00000048217 | -0.44 | -0.39 | 0.73 | 0.47 | 0.28 | 0.33 | 0.31 | 0.20 |
| ASNS | ENSMUSG00000029752 | 0.55 | 0.02 | 0.02 | 0.40 | 0.43 | 0.32 | -0.03 | 0.11 |
| DHRS9 | ENSMUSG00000027068 | 0.22 | -0.28 | 0.31 | 0.66 | 0.35 | 0.38 | 0.27 | 0.11 |
| HAL | ENSMUSG00000020017 | -0.06 | 0.01 | 0.31 | 0.42 | 0.22 | 0.21 | -0.05 | -0.17 |
| RAB8B | ENSMUSG00000036943 | 0.09 | 0.16 | 0.22 | 0.30 | -0.22 | 0.30 | 0.15 | 0.02 |
| **CTSS** | **ENSMUSG00000038642** | **-0.05** | **0.18** | **0.31** | **0.21** | **-0.12** | **0.45** | **-0.03** | **0.17** |
| **CD14** | **ENSMUSG00000051439** | **0.46** | **0.46** | **0.41** | **0.36** | **0.14** | **0.69** | **0.16** | **0.03** |
| LMNB1 | ENSMUSG00000024590 | 0.13 | 0.31 | 0.33 | 0.22 | 0.33 | 0.16 | 0.29 | 0.13 |
| **NCF4** | **ENSMUSG00000071715** | **-0.08** | **0.23** | **0.38** | **0.37** | **0.21** | **0.47** | **0.11** | **0.07** |
| **VAV1** | **ENSMUSG00000034116** | **-0.10** | **0.18** | **0.47** | **0.37** | **0.21** | **0.47** | **0.28** | **0.15** |
| **PSMB9** | **ENSMUSG00000024337** | **-0.30** | **0.17** | **0.38** | **0.58** | **0.35** | **0.25** | **0.43** | **0.12** |
| **PSMB8** | **ENSMUSG00000024338** | **-0.22** | **0.26** | **0.45** | **0.66** | **0.37** | **0.35** | **0.46** | **0.16** |
| **CD180** | **ENSMUSG00000021624** | **-0.10** | **0.45** | **0.61** | **0.67** | **0.24** | **0.30** | **0.44** | **0.06** |
| HIST1H1D | ENSMUSG00000052565 | 0.00 | 0.42 | 0.56 | 0.37 | 0.30 | 0.26 | 0.71 | 0.10 |
| **C1R** | **ENSMUSG00000092005** | **-0.09** | **0.11** | **0.32** | **0.48** | **-0.11** | **0.74** | **0.51** | **0.20** |
| SAMHD1 | ENSMUSG00000027639 | -0.07 | 0.29 | 0.35 | 0.51 | 0.13 | 0.47 | 0.52 | 0.26 |
| **TNFSF8** | **ENSMUSG00000028362** | **0.13** | **0.55** | **0.45** | **0.56** | **0.46** | **-0.04** | **0.40** | **0.16** |
| **AIF1** | **ENSMUSG00000024397** | **-0.04** | **0.47** | **0.71** | **0.32** | **0.25** | **0.27** | **0.14** | **0.09** |
| CD72 | ENSMUSG00000028459 | -0.05 | 0.36 | 0.83 | 0.35 | 0.42 | 0.41 | 0.24 | 0.22 |
| FAM54A | ENSMUSG00000019992 | 0.02 | 0.39 | 0.79 | 0.08 | 0.47 | 0.30 | 0.50 | 0.21 |
| SGOL1 | ENSMUSG00000023940 | 0.10 | 0.47 | 0.74 | 0.29 | 0.62 | 0.25 | 0.39 | 0.18 |
| **LAIR1** | **ENSMUSG00000055541** | **-0.13** | **0.17** | **0.44** | **0.51** | **0.57** | **0.66** | **0.44** | **0.49** |
| **BID** | **ENSMUSG00000004446** | **0.12** | **0.19** | **0.34** | **0.75** | **0.52** | **0.28** | **0.17** | **-0.02** |
| **TRIM5** | **ENSMUSG00000057143** | **-0.03** | **0.32** | **0.45** | **0.92** | **0.45** | **0.31** | **-0.02** | **-0.30** |
| **SIGLEC1** | **ENSMUSG00000027322** | **0.05** | **0.52** | **0.66** | **0.76** | **0.62** | **0.32** | **-0.13** | **-0.17** |
| B3GNT3 | ENSMUSG00000031803 | -0.28 | 0.35 | 0.39 | 0.90 | 0.85 | 0.37 | -0.01 | 0.05 |
| FFAR2 | ENSMUSG00000051314 | 0.18 | 0.20 | 0.33 | 1.13 | 0.85 | 0.45 | -0.07 | -0.03 |
| **IFIH1** | **ENSMUSG00000026896** | **0.03** | **0.63** | **0.52** | **1.02** | **0.56** | **0.35** | **0.29** | **-0.24** |
| TAP1 | ENSMUSG00000037321 | -0.09 | 0.50 | 0.69 | 0.81 | 0.53 | 0.51 | 0.28 | 0.09 |
| **STAT1** | **ENSMUSG00000026104** | **-0.07** | **0.62** | **0.76** | **0.78** | **0.56** | **0.26** | **0.49** | **0.00** |
| RTP4 | ENSMUSG00000033355 | 0.08 | 0.72 | 0.70 | 0.78 | 0.26 | 0.48 | 0.49 | -0.13 |
| XAF1 | ENSMUSG00000040483 | 0.06 | 0.72 | 0.71 | 0.96 | 0.77 | 0.65 | 0.54 | -0.28 |
| **CD274** | **ENSMUSG00000016496** | **0.02** | **0.60** | **0.66** | **1.43** | **1.10** | **0.25** | **0.76** | **0.17** |
| OAS2 | ENSMUSG00000032690 | 0.24 | 0.94 | 0.93 | 1.45 | 0.85 | 0.45 | 0.46 | -0.35 |
| **IFIT2** | **ENSMUSG00000045932** | **0.31** | **1.03** | **0.95** | **1.48** | **0.74** | **0.43** | **0.55** | **-0.23** |
| USP18 | ENSMUSG00000030107 | 0.23 | 0.99 | 0.99 | 1.73 | 1.02 | 0.40 | 0.38 | -0.28 |
| SAA4 | ENSMUSG00000040017 | -0.91 | -0.43 | 0.19 | 0.98 | 0.92 | 1.43 | 0.61 | 0.63 |
| **NMU** | **ENSMUSG00000029236** | **0.19** | **0.08** | **0.41** | **1.39** | **1.30** | **0.92** | **0.66** | **0.91** |
| SFN | ENSMUSG00000047281 | 0.53 | 0.18 | 0.26 | 0.72 | 1.06 | 1.31 | 0.94 | 0.90 |
| OASL | ENSMUSG00000041827 | 0.55 | 1.47 | 1.29 | 1.77 | 1.26 | 1.21 | 1.50 | 0.33 |
| **CXCL10** | **ENSMUSG00000034855** | **0.85** | **1.89** | **1.61** | **1.95** | **1.01** | **0.77** | **1.40** | **0.33** |
|  |  |  |  |  |  |  |  |  |  |

Average log10(ratio) gene expression of 53 DE genes commonly differentially expressed in the lung of mice, macaque and swine infected with CA04 at each day p.i. Swine and mouse CA04-infected lung gene expression is referenced against time-matched, species-matched mock-infected lung gene expression. Macaque CA04-infected lung gene expression is referenced against species-matched uninfected lung gene expression. The bolded genes are associated with the inflammatory response.
