# Supplementary material for: 2009 pandemic H1N1 influenza virus elicits similar clinical course but differential host transcriptional response in mouse, macaque, and swine infection models
Source: BMC Genomics. 2012 Nov 15;13:627. doi: 10.1186/1471-2164-13-627 (PMC3532173; doi:10.1186/1471-2164-13-627)
Supplement: Additional file 7 — Table S4. Gene expression of LXR/RXR and VDR/RXR genes in mouse, macaque and swine lung infected with CA04 virus. (DOC 69 kb) [file 1471-2164-13-627-S7.doc]

| **Supplementary Table 4**. Gene expression of LXR/RXR and VDR/RXR genes in mouse, macaque and swine lung infected with CA04 virus | | | | | | | | | |
| --- | --- | --- | --- | --- | --- | --- | --- | --- | --- |
| **Gene Symbol** | **Ensembl Gene ID** | ***Mouse*** | | | ***Macaque*** | | ***Swine*** | | |
|  |  | **1 dpi** | **3 dpi** | **5 dpi** | **1 dpi** | **6 dpi** | **3 dpi** | **5 dpi** | **7 dpi** |
|  |  |  |  |  |  |  |  |  |  |
| Alb | ENSMUSG00000029368 | -2.42 | -2.63 | -2.12 | 0.04 | 0.06 | -0.25 | 0.05 | -2.42 |
| Apoc3 | ENSMUSG00000032081 | -2.61 | -2.20 | -1.42 | 0.78 | 0.74 | -0.20 | -0.18 | -2.61 |
| Apoa1 | ENSMUSG00000032083 | -2.08 | -2.47 | -1.30 | 0.61 | 0.47 | 0.30 | -0.07 | -2.08 |
| Apoh | ENSMUSG00000000049 | -2.27 | -2.35 | -1.41 | -0.30 | -0.04 | 0.47 | 0.62 | -2.27 |
| Ambp | ENSMUSG00000028356 | -2.11 | -2.27 | -0.82 | -0.09 | -0.38 | -0.08 | -0.06 | -2.11 |
| Apof | ENSMUSG00000047631 | -1.98 | -1.58 | -0.64 | -0.07 | -0.06 | -0.01 | 0.29 | -1.98 |
| Kng1 | ENSMUSG00000022875 | -1.89 | -1.58 | -0.55 | 0.54 | 0.48 | 0.51 | 0.41 | -1.89 |
| **Igfbp1** | **ENSMUSG00000020429** | **-0.68** | **-1.31** | **-0.03** | **-1.81** | **-0.92** | **-0.10** | **-0.20** | **-0.68** |
| **Hsd17b2** | **ENSMUSG00000031844** | **-0.24** | **-0.04** | **0.49** | **-1.33** | **-0.74** | **-0.05** | **-0.26** | **-0.24** |
| **Camp** | **ENSMUSG00000038357** | **-0.47** | **-0.27** | **0.02** | **-0.12** | **-0.45** | **-0.47** | **1.04** | **-0.47** |
| Pon1 | ENSMUSG00000002588 | -1.25 | -0.92 | -0.73 | -0.16 | -0.07 | 0.29 | -0.33 | -1.25 |
| Apob | ENSMUSG00000020609 | -1.04 | -1.14 | -0.26 | -0.04 | 0.03 | 0.11 | 0.02 | -1.04 |
| Hpx | ENSMUSG00000030895 | -1.24 | -0.82 | 0.06 | 0.48 | 0.45 | -0.08 | 0.01 | -1.24 |
| Cyp7a1 | ENSMUSG00000028240 | -0.56 | -0.69 | -0.22 | 0.00 | 0.14 | -0.41 | -0.49 | -0.56 |
| Apom | ENSMUSG00000024391 | -0.59 | -0.68 | -0.26 | -0.14 | -0.03 | -0.14 | 0.05 | -0.59 |
| Apoc2 | ENSMUSG00000002992 | -0.47 | -0.19 | 0.34 | 0.07 | -0.06 | -0.29 | -0.22 | -0.47 |
| C9 | ENSMUSG00000022149 | -0.66 | -0.81 | 0.02 | 0.16 | 0.18 | 0.51 | 0.54 | -0.66 |
| **Calb1** | **ENSMUSG00000028222** | **-0.38** | **-0.16** | **-0.11** | **-0.64** | **-0.66** | **0.26** | **-0.08** | **-0.38** |
| **Igfbp5** | **ENSMUSG00000026185** | **-0.18** | **-0.18** | **-0.25** | **-0.42** | **-0.42** | **-0.11** | **-0.28** | **-0.18** |
| Tf | ENSMUSG00000032554 | -0.32 | -0.19 | -0.09 | -0.31 | -0.12 | -0.01 | -0.18 | -0.32 |
| Cd36 | ENSMUSG00000002944 | -0.04 | -0.10 | -0.30 | -0.25 | 0.03 | 0.29 | -0.05 | -0.04 |
| **Ncoa2** | **ENSMUSG00000005886** | **-0.01** | **0.01** | **0.02** | **-0.05** | **-0.40** | **0.11** | **0.07** | **-0.01** |
| Lpl | ENSMUSG00000015568 | -0.08 | -0.04 | -0.13 | -0.05 | -0.45 | 0.53 | -0.16 | -0.08 |
| Nr1h3 | ENSMUSG00000002108 | -0.07 | 0.01 | 0.03 | 0.05 | 0.06 | 0.30 | -0.09 | -0.07 |
| C3 | ENSMUSG00000024164 | -0.03 | 0.06 | 0.23 | 0.28 | -0.02 | 0.44 | 0.10 | -0.03 |
| Apoe | ENSMUSG00000002985 | -0.27 | -0.26 | 0.01 | 0.04 | 0.14 | 0.45 | 0.05 | -0.27 |
| Abca1 | ENSMUSG00000015243 | -0.16 | -0.17 | -0.13 | 0.12 | 0.07 | 0.49 | 0.33 | -0.16 |
| Ly96 | ENSMUSG00000025779 | -0.08 | -0.03 | 0.06 | 0.43 | 0.28 | 0.56 | 0.26 | -0.08 |
| Abcg8 | ENSMUSG00000024254 | 0.36 | -0.13 | 0.09 | 0.17 | 0.35 | -0.31 | -0.26 | 0.36 |
| **Rxrg** | **ENSMUSG00000015843** | **-0.02** | **0.09** | **0.03** | **0.49** | **0.40** | **-0.08** | **0.12** | **-0.02** |
| **Hr** | **ENSMUSG00000022096** | **-0.01** | **0.08** | **0.12** | **0.33** | **0.30** | **0.11** | **-0.10** | **-0.01** |
| **Il2** | **ENSMUSG00000027720** | **0.06** | **-0.05** | **0.13** | **0.41** | **0.50** | **0.05** | **-0.06** | **0.06** |
| **Cyp24a1** | **ENSMUSG00000038567** | **0.28** | **0.10** | **0.16** | **0.84** | **0.63** | **-0.10** | **-0.56** | **0.28** |
| Tnf | ENSMUSG00000024401 | 0.07 | 0.61 | 0.46 | 0.15 | -0.10 | 0.03 | -0.02 | 0.07 |
| Clu | ENSMUSG00000022037 | 0.15 | 0.38 | 0.34 | 0.01 | -0.07 | 0.71 | 0.31 | 0.15 |
| **Cd14** | **ENSMUSG00000051439** | **0.46** | **0.46** | **0.41** | **0.36** | **0.14** | **0.69** | **0.16** | **0.46** |
| Msr1 | ENSMUSG00000025044 | 0.28 | 0.62 | 0.89 | 0.11 | 0.19 | 0.39 | 0.13 | 0.28 |
| Apod | ENSMUSG00000022548 | 0.23 | 0.88 | 1.07 | 0.15 | 0.08 | 0.45 | 0.54 | 0.23 |
| Saa4 | ENSMUSG00000040017 | -0.91 | -0.43 | 0.19 | 0.98 | 0.92 | 1.43 | 0.61 | -0.91 |
| Il6 | ENSMUSG00000025746 | 1.43 | 1.65 | 1.05 | 2.00 | 1.40 | 0.21 | 0.50 | 1.43 |
| Ccl2 | ENSMUSG00000035385 | 0.83 | 1.66 | 1.43 | 1.23 | 1.02 | 0.57 | 0.73 | 0.83 |
| **Cxcl10** | **ENSMUSG00000034855** | **0.85** | **1.89** | **1.61** | **1.95** | **1.01** | **0.77** | **1.40** | **0.85** |
|  |  |  |  |  |  |  |  |  |  |

Average log10(ratio) gene expression in the lung of mice, macaque and swine infected with CA04 at each day p.i. Swine and mouse gene expression is referenced against time-matched, species-matched mock-infected lungs. Macaque gene expression is referenced against species-matched mock-infected lungs. The bolded genes are associated with VDR/RXR Activation.
