# Supplementary material for: 2009 pandemic H1N1 influenza virus elicits similar clinical course but differential host transcriptional response in mouse, macaque, and swine infection models
Source: BMC Genomics. 2012 Nov 15;13:627. doi: 10.1186/1471-2164-13-627 (PMC3532173; doi:10.1186/1471-2164-13-627)
Supplement: Additional file 8 — Table S5. Summary of CA04 induced DE gene sets for each species. (DOC 30 kb) [file 1471-2164-13-627-S8.doc]

| **Supplementary Table 5**. Summary of CA04 induced DE gene sets for each species | | | |
| --- | --- | --- | --- |
| **Animal model** | **DE genes** | **Mapped IDs** | **Unmapped IDs** |
| *Mouse* | 697 | 696 | 1 |
| *Macaque* | 771 | 771 | 0 |
| *Swine* | 616 | 611 | 5 |

DE genes in response to CA04 virus were defined by Student’s t-test (*P* < 0.05 on at least one day) and as having average FC ≥ 2 relative to species-matched mock. Gene expression data were uploaded into Ingenuity Pathway Analysis for functional analysis. Human annotations were used for macaque and swine gene IDs. The number of gene IDs mapping to known identifiers in the IPKDB are shown.
